# Topical NAVS naphthalan for the treatment of oral lichen planus and recurrent aphthous stomatitis: A double blind, randomized, parallel group study

**DOI:** 10.1371/journal.pone.0249862

**Published:** 2021-04-08

**Authors:** Ana Andabak Rogulj, Iva Z. Alajbeg, Vlaho Brailo, Ivana Škrinjar, Ivona Žužul, Vanja Vučićević-Boras, Ivan Alajbeg

**Affiliations:** 1 Department of Oral Medicine, School of Dental Medicine, University of Zagreb, Zagreb, Croatia; 2 Department of Dentistry, Clinical Hospital Center Zagreb, Zagreb, Croatia; 3 Department of Removable Prosthodontics, School of Dental Medicine, University of Zagreb, Zagreb, Croatia; 4 Department of Oral Surgery, School of Dental Medicine, University of Zagreb, Zagreb, Croatia; State University of Ponta Grossa, BRAZIL

## Abstract

**Aim:**

To evaluate the effectiveness of non-aromatic very rich in steranes (NAVS) naphthalan in the treatment of oral lichen planus (OLP) and recurrent aphthous stomatitis (RAS). Null hypothesis was that there would be no difference between NAVS and topical steroids in the treatment of OLP and RAS.

**Methods:**

The study consisted of two sub-trials conducted as randomized, double-blind controlled studies: first included OLP patients and second patients with RAS. Patients received either NAVS or 0.05% betamethasone dipropionate. Primary outcomes were activity score (OLP patients), No of lesions and lesion diameter (RAS patients) and pain intensity (VAS) while secondary outcome included the impact of the disease on quality of life assessed by Oral health impact profile (OHIP 14).

**Results:**

No significant differences in terms of OLP clinical signs (p = 0.84, η^2^ = 0.001) and responses on the OHIP-14 (p = 0.81, η^2^ = 0.002) or on VAS (p = 0.14, η^2^ = 0.079) between NAVS and betamethasone groups were observed. In RAS patients, no significant differences between the groups in terms of lesion number (at days 3 and 5, *p* = 0.33 and *p* = 0.98, respectively), lesion diameter (days 3 and 5, *p* = 0.24 and *p* = 0.84, respectively) were observed. However, in NAVS group a significant reduction of lesions diameter was observed on the 3^rd^ day, while in betamethasone group a significant reduction in lesions diameter was evident only after the 5^th^ day. No significant differences in VAS (p > 0.05) and the OHIP-14 (p > 0.05) between groups were found.

**Conclusion:**

No evidence of differences between the two compared interventions was found.

**Registration:**

Retrospective registration of this trial was conducted in ClinicalTrials.gov on September 30, 2016; trial registration number: NCT02920658.

https://clinicaltrials.gov/ct2/show/NCT02920658?term=NAVS&draw=2&rank=4.

## Introduction

Non-aromatic-very rich in steranes (NAVS) naphthalan is a transparent, earth mineral oil prepared by a complex set of separations and refining steps. The process begins with an oil that is used as the starting material for brown naphthalan, which has been successfully used in the treatment of psoriasis [1, 2]. We used liquid chromatography to remove potentially mutagenic polycyclic aromatic hydrocarbons (PAHs). UV / VIS spectrophotometry confirmed that PAHs were below the detection threshold [3]. Since steranes contain similar chemical structures as well-known bioactive substances, such as vitamin D3 and steroid hormones, we proposed that NAVS is effective in the treatment of immune-mediated oral diseases, such as OLP and RAS. To date, NAVS has been extensively investigated *in vitro* and *in vivo* studies in animals and humans [2, 4]. Naphthalan has antiproliferative effects and reduces the number of immunocompetent cells in psoriatic skin [5]. Naphthalan *in vitro* appears to inhibit proliferation of keratinocytes, with a tendency towards normalization in psoriatic skin [6]. Another study showed pronounced dose-dependent inhibition of proliferation in a squamous cell carcinoma cell line. However, naphthalan does not inhibit non-malignant fibroblasts, indicating its selectivity in cell growth control [1]. In a mouse model of squamous cell carcinoma, naphthalan slowed tumor neoangiogenesis [7]. Many years of monitoring patients on naphthalan therapy showed no disturbance in hematological or biochemical profiles [2, 4]. Topical steroids are considered first-line therapy for many chronic immune-mediated oral diseases. Risks of short-term use of topical corticosteroids are clinically insignificant. However, long-term use is not recommended because of potential side effects, such as mucosal atrophy, secondary infection with *Candida albicans*, possible systemic absorption, and adrenal suppression [8]. Previously, we published a pilot study using topical NAVS naphthalan and the results were favorable [9]. Our null hypothesis was that there would be no difference between NAVS and topical steroids in the treatment of OLP and RAS.

## Materials and methods

**Fig 1:**
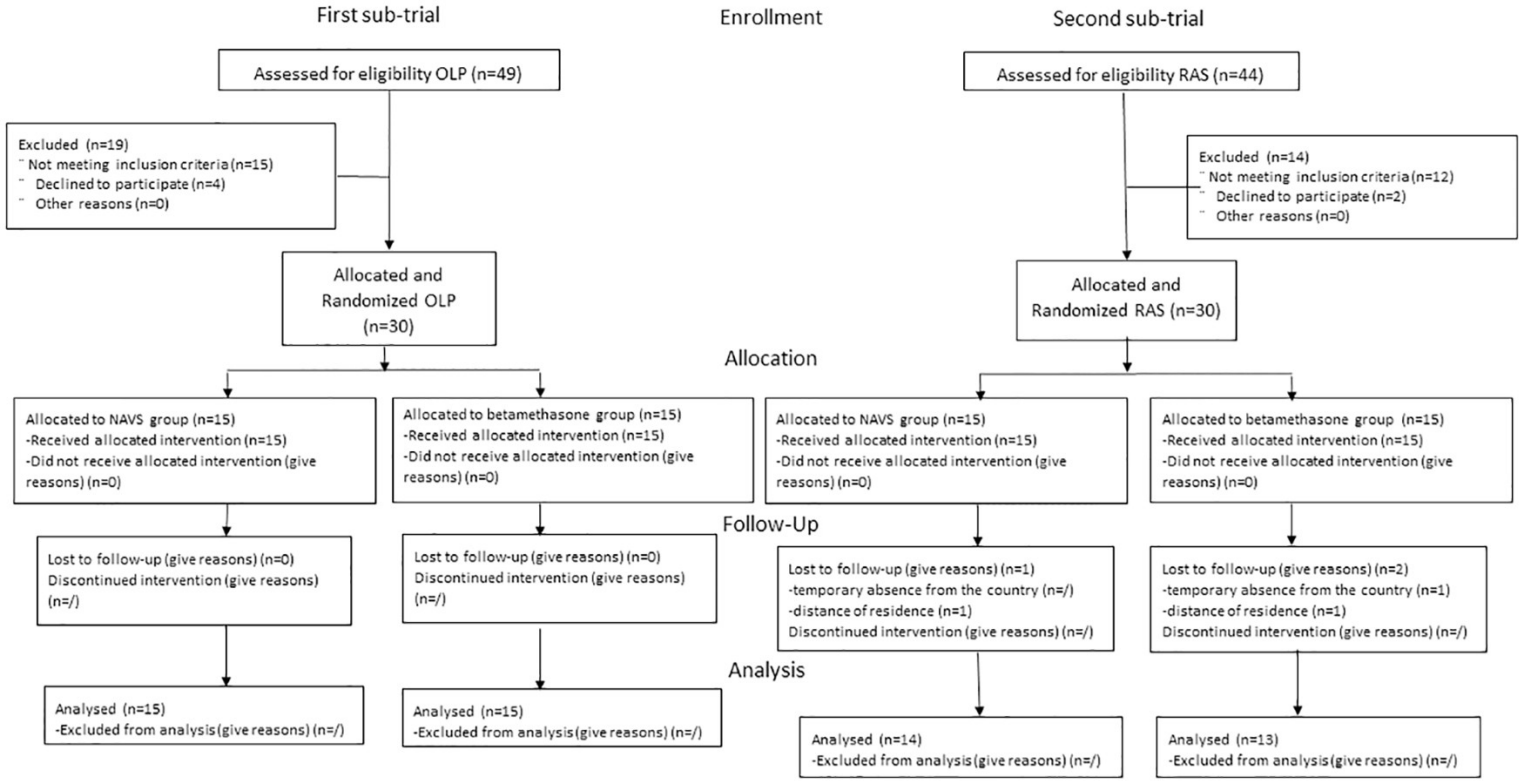
CONSORT diagram.

The study was approved by the Ethics Committee of the School of Dental Medicine, University of Zagreb and it consisted of two sub-trials: first included OLP patients and second patients with RAS. Both sub-trials were conducted as a randomized, double-blind controlled studies (participants were randomized into two groups: NAVS and betamethasone) with intended allocation ratio of 1:1. The final number of subjects was 30 for the OLP sub-trial and 27 for the RAS sub-trial. Both sub-trials were designed as parallel group, superiority studies. All participants gave informed written consent. Retrospective registration of this trial was done at ClinicalTrials.gov on September 30, 2016. During the time this trial was conducted (between December 2010 and November 2013), registering trials at international registries was not considered mandatory by every journal. Registering trials at our IRB was the accepted procedure at that time, and we were not aware about today’s preferable and improved policy, which we have corrected. The authors confirm that all ongoing and related trials for this drug/intervention are registered. The CONSORT diagram (Fig 1) shows allocation per study group.

### Eligibility criteria

Study participants were adult patients of the Department of Oral Medicine, School of Dental Medicine in Zagreb, with clinically and histologically proven OLP [10], or RAS (at least 2 episodes per year) with daily subjective symptoms in the acute stage [11]. Exclusion criteria for OLP patients were as follows: age < 18 years; hepatobiliary system disease; lichenoid reaction (amalgam, drugs) or lichen planus with lesions in contact with restorative materials [12]; current systemic or local anti-inflammatory treatment (antibiotics, corticosteroids, non-steroidal anti-rheumatic drugs, chemotherapeutics) [8, 13, 14]; and pregnancy. Exclusion criteria for RAS patients were as follows: age < 18 years; hematological deficits (assessed by complete blood count, iron, and vitamin B12); hypersensitivity to toothpaste and oral mouth rinse solutions (assessed by medical history) [13]; pregnancy; inflammatory bowel disease; significant immunodeficiency; and current systemic or topical anti-inflammatory treatment (antibiotics, corticosteroids, antimycotics, nonsteroidal antirheumatics, and chemotherapeutics) [8, 13, 14].

### Study setting

This was a single center study carried out at an outpatient clinic of a major Croatian tertiary academic center.

### Interventions

NAVS naphthalan was produced from a Croatian natural oil as described in [1]. We prepared the experimental compound by mixing NAVS oil and adhesive powder (Stomahesive^®^, ConvaTec, Deeside, Flintshire, UK) in a volume ratio of 2:1. The control group was treated with 0.05% betamethasone dipropionate ointment (Beloderm^®^, Belupo, Koprivnica, Croatia) in the same adhesive paste (1:1). Participants were instructed to dry affected mucosa with a gauze, apply one of the therapeutic agents using a cotton swab and refrain from eating and drinking for half an hour. Application of the therapeutic agent was performed three times daily for 4 weeks for OLP patients and three times daily for 7 days for RAS patients.

### OLP patients

#### Primary outcome measures

*Activity score*. For OLP patients, we measured clinical improvement and subjective symptomatic relief. The severity of OLP lesions was scored as described [15] at days 0 and 28, via photographs. This clinical scale measures the presence of reticular, erythematous and ulcerative lesions (REU) on oral mucosal surfaces, and generates a score by adding those values. Three examiners independently reviewed and evaluated each photograph. Photographs were evaluated for the second time one week later to validate the first reading. Results were analyzed using Spearman “rank” correlation to determine intra- and inter-observer reliability [15].

*The intensity of pain*. For estimating subjective symptomatic improvement, the intensity of pain and discomfort was determined using a 100-mm visual analog scale (VAS) before and after 28 days of therapy [16, 17].

#### Secondary outcome measure

*Oral Health Impact Profile (OHIP-14)*. The short-form OHIP-14 questionnaire, translated and validated, was used to show how clinical oral outcomes impact patients’ quality of life before and after 28 days of therapy [16, 17].

Patients were followed until the 8^th^ week after the initiation of the treatment to record possible relapses.

### RAS patients

#### Primary outcome measures

*Number and size of lesions*. We measured clinical outcomes by the decrease in number and size of lesions, and by patients’ symptom reports during the treatment period. The number and the diameter of RAS lesions were assessed on days 0, 3 and 5 [18].

*The intensity of pain*. Pain intensity was determined using a visual analog scale (VAS) on day 0 before application, and every day after the application at home, and recorded in a pain diary for one week. For estimating subjective symptomatic improvement, the intensity of pain before application of NAVS or betamethasone (day 0) and on day 3 and day 5 of therapy was compared.

#### Secondary outcome measures

*Oral Health Impact Profile (OHIP-14)*. The impact of the disease on quality of life was assessed by the OHIP 14 questionnaire on days 0 and 8.

Secondary outcome measures also included side-effects of treatment. We wanted to assess tolerability of the usage of NAVS and therefore we compared it to betamethasone with the use of VAS. For that matter, we have instructed participants to record VAS 30 minutes and 60 minutes following each application. Recordings of middle daily application (the 2nd application) were used for comparison between groups.

*Sample size determination*. We performed a statistical power analysis for sample size estimation in OLP patients based on data from our pilot study [9] and the study of Hegarty et al [19]. The administration of NAVS for 28 days [9] resulted in 52.2% overall clinical improvement of cumulative activity scores while after 6 weeks of therapy there was a 41% reduction in the mean total surface area of oral lesions in the patient group using betamethasone [19]. A sample size calculation for a repeated measurement within-between analysis of variance with two groups showed that 24 participants (12 per group) were required to obtain a power of 0.80 at an alpha level of 0.05. Thus, our proposed sample size of 30 OLP patients should be adequate for the main objective of this study.

The sample size calculation for sample size estimation in RAS patients was modeled from our pilot study conducted as compared double blind randomized (topical betamethasone in adhesive paste used as control) [9]. Power analyses based upon the percentage of the number of residual aphthous lesions on day (25%, 44%, respectively, estimated SD 18) revealed that 24 participants (12 per group) were necessary to achieve 80% power with a significance level of 0.05. Thus, our proposed sample size of 30 RAS patients should be adequate for the main objective of this study.

### Allocation

We divided subjects into two equal groups using block randomization, with block sizes of two and four, randomly mixed. Investigators were blinded to sizes of each block [20].

One member of the team (a nurse), who did not evaluate the therapeutic effect, allocated participants to test and control preparations in accordance with the randomization list. Facts that randomization allocation block sizes have been randomly changing, as well as that the nurse was not trained to assess clinical severity of oral disease, have attributed to decrease of the allocation bias.

### Blinding

Identical sealed containers containing either of treatments were given to the participants. The appearance and consistency of both preparations were indistinguishable. Participants were unaware of the differences of each intervention. Two oral medicine specialists who assessed therapeutic outcomes were blinded to allocation sequences and to treatment modalities.

### Statistical methods

Analyses were performed using Statistica 13.4.0 software package (1984–2018 TIBCO Software Inc.). The distribution of data was tested using the Shapiro-Wilk test. A series of independent sample t-tests were carried out first in order to determine whether there were any differences between the two randomized groups (NAVS, betamethasone) both for OLP and RAS patients at baseline. Additional analyses were performed out to assess whether outcome variables were correlated significantly at baseline. Fisher exact test was used to compare gender representation among the groups.

By using ANCOVA the differences between groups were tested in order to determine whether the outcome variables during follow-up appointments, adjusted for baseline scores, differ between the two treatment groups (NAVS, betamethasone) both for OLP and RAS patients. Baseline variables i.e., activity score for OLP patients, No of lesions and lesion diameter for RAS patients as well as pain intensity and OHIP score both for OLP and RAS patients were included as covariates.

The Within subjects repeated measures ANOVA was used to test the within-group changes for NAVS and betamethasone in primary and secondary treatment outcomes at follow-up appointments, both for OLP and RAS patients. Bonferroni corrected post hoc tests were used to evaluate the difference between the time points. Partial eta squared (η^2^) was used as a measure of the effect sizes.

If the sphericity assumption was violated (Mauchly’s test of sphericity p<0.05), Greenhouse-Geisser corrections were performed.

The percentage change of primary and secondary outcome values was analyzed by means of independent sample t-tests.

## Results

We recruited patients between December 2010 and November 2013. Forty nine OLP and forty four RAS patients were assessed for eligibility. Nineteen OLP and fourteen RAS patients were excluded from the study. In a first sub-trial a total of 30 OLP patients were randomized, while in second sub-trail a total of 30 RAS patients were randomized. Three RAS patients were lost at the follow-up due to temporary absence from the country (n = 1) and distant residence (n = 2). Thirty patients in first sub-trial and 27 patients in second sub-trial completed the study. See Fig 1 for CONSORT diagram.

### OLP patients

Results from the analysis of inter- and intra-observer reliability are displayed in Table 1. Three calibrated investigators used an OLP scoring system [15] for both photographic evaluations at seven day intervals. The Spearman correlation coefficient was 0.96 for intra-observer agreement. Inter-observer agreement for evaluation of three calibrated investigators showed high correlation before (0.99) and after the therapy (0.98).

**Table 1:** Data from the analysis of intra- and inter-observer variability.

	Intra-observer						Inter-observer		
	Investigator I		Investigator II		Investigator III		Investigator I, II, III		Investigator I, II, III
**assessment**	1.	2.	1.	2.	1.	2.	1.		2.
**rho**	0.985	0.958	0.944	0.902	0.980	0.911	ICC	0.9860	0.9797
**CI (95%)**	0.969–0.993	0.913–0.980	0885–0.973	0.802–0.953	0.958–0991	0.821–0.957	0.9744–0.9929		0.9628–0.9897

Spearman’s coefficient of rank correlation (rho).

ICC- the degree of consistency among measurements.

CI—estimates the reliability of averages of investigators ratings.

Thirty OLP patients (26 women and 4 men) were enrolled in the first sub-trail. All patients completed the study. Demographics and clinical features are displayed in Table 2. There were no significant differences between the groups with respect to age (*p* = 0.441) and gender (*p* = 1.0). Treatment groups did not differ at baseline on either primary or secondary outcomes (activity score *p* = 0.523; VAS *p* = 0.189, OHIP-14 *p* = 0.693). As expected, several variables were significantly correlated at baseline. Activity score was significantly correlated with both VAS (r = 0.765, p < 0.001) and OHIP-14 (r = 0.406, p = 0.026).

**Table 2:** Demographics and clinical features of the OLP patients (NAVS—“Non-aromatic very rich in steranes naphthalan).

	NAVS (N = 15)	Betamethasone (N = 15)
**Atrophic OLP**	10	11
**Erosive OLP**	5	4
**Gender (M/F)**	2/13	2/13
**Age (years), mean ± SD**	63 ± 8.36	65 ± 9.19

#### Clinical improvement

**Fig 2:**
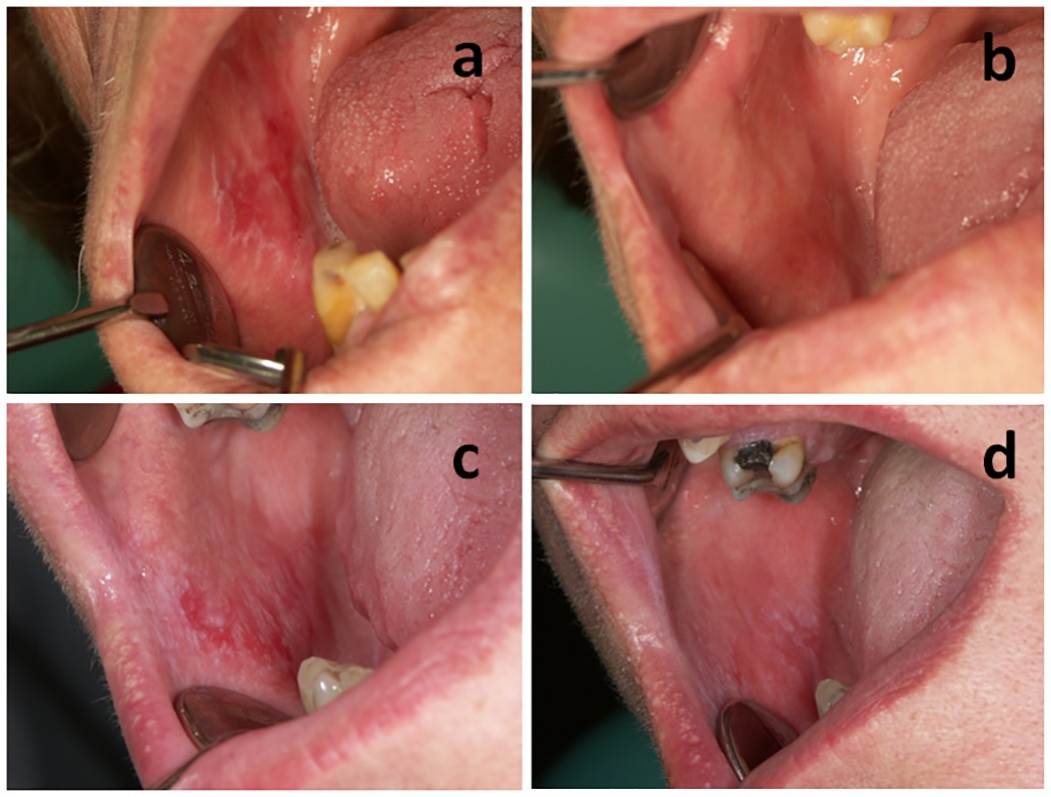
Patient with OLP on day 0 (a) and on day 28 (b) of betamethasone treatment, and on day 0 (c) and on day 28 (d) of NAVS treatment.

*Primary outcome measures changes*. While controlling for baseline values, at 28^th^ day follow-up patients in both treatment groups demonstrated reduction of activity score (Table 4) but the differences between the groups were not statistically significant (Table 3). Example of a patient with OLP on day 0 and on day 28 of betamethasone treatment is presented in Fig 2a and 2b, while the example of a patient with OLP on day 0 and on day 28 of NAVS treatment is presented in Fig 2c and 2d.

**Table 3:** Means and SDs of outcome measures for each group at each time point (OLP patients).

		NAVS		Betamethasone		Partial eta-squared^ф^
		Baseline	28^th^ day follow-up	Baseline	28^th^ day follow-up	Baseline vs 28^th^ day follow-up
**Primary outcomes**	*activity score*	8.13 (4.29)	2.66 (1.89)	9.2 (4.73)	3.1 (2.44)	0.001
	*pain intensity*	27 (14.5)	1 (1.25)	34.73 (16.9)	2.20 (2)	0.079
**Secondary outcome**	*OHIP score*	21.26 (8.53)	7.2 (4.44)	19.93 (9.75)	7 (4.47)	0.002

^ф^ Effect size for treatment differences between NAVS and Betamethasone group.

OLP cumulative activity score in the NAVS group on days 0 and 28 were 122 and 40, respectively (*p* = 0.0001). Using NAVS for 28 days resulted in 66.55% overall clinical improvement. OLP cumulative activity score in the betamethasone group on days 0 and 28 were 138 and 46.5, respectively (*p* = 0.0001). Using 0.05% betamethasone dipropionate for 28 days resulted in 66.03% overall clinical improvement. There were no significant differences between the groups (*p* = 0.935).

Within-group analyses (Table 4) showed a significant decrease in pain intensity both for NAVS and betamethasone group. However, the differences between the groups were not statistically significant (Table 3).

**Table 4:** Within groups ANOVA—Post-hoc pairwise comparisons (OLP pateints).

			NAVS			Betamethasone		
			Mean differ.	95% CI	p ^ᵻ^	Mean differ.	95% CI	p ^ᵻ^
**Primary outcomes**	*activity score*	Baseline vs 28^th^ day follow-up	5.46	3.44–7.49	**<0.0001**	6.1	3.99–8.21	**<0.0001**
	*pain intensity*	Baseline vs 28^th^ day follow-up	26.00	18.24–33.76	**<0.0001**	32.53	23.45–41.62	**<0.0001**
**Secondary outcome**	*OHIP score*	Baseline vs 28^th^ day follow-up	14.07	10.42–17.71	**<0.0001**	12.93	9.29–16.57	**<0.0001**

CI: Confidence Interval;

^ᵻ^bolded p-values represent statistically significant differences.

Cumulative scores on VAS in the NAVS group on days 0 and 28 were 405 and 15, respectively, representing a reduction of 96.41%. Cumulative scores on VAS in the betamethasone group on days 0 and 28 were 521 and 33, respectively, a reduction of 93.24%. No significant differences between groups (*p* = 0.21) were found.

*Secondary outcome measures changes*. Improvement in OHIP-14 score at 28-day follow-up was evident both in NAVS and in betamethasone group (Table 4) but did not achieve statistical significance at the 0.05 level (Table 3).

When comparing changes in secondary outcome between NAVS and betamethasone, the relative decrease was not significantly different (67.12, 65.74%, respectively; *p* = 0.81).

#### Side effects

Oral candidiasis was recorded in three patients, who were treated with betamethasone, during the 4^th^ week of treatment. It was diagnosed by clinical presentation and positive candida culture. Topical 2% miconazole gel was administered, leading to resolution of the infection. Other adverse reactions were not reported. There were no relapses recorded over 8 weeks in either group.

### RAS patients

In the second sub-trial 30 RAS patients (17 women and 13 men) were enrolled. Twenty-seven subjects (14 women and 13 men) completed the study. After opening the randomization code, we found that 14 patients used NAVS and 13 used betamethasone.

Experimental and control groups were well matched with respect to age and gender (age, *p* = 0.29; gender, *p* = 0.3389). The mean age was 48.01 ± 17,45. Treatment groups did not differ at baseline on either primary or secondary outcomes (number of lesions, *p* = 0.64; lesion diameter, *p* = 0.82, VAS *p* = 0.36, OHIP-14 *p* = 0.184). Variables that were significantly correlated at baseline were lesion number and VAS (r = 0.438, p = 0.015).

Demographics and clinical features are displayed in Table 5.

**Table 5:** Demographics and clinical features of the RAU patients (NAVS—“Non-aromatic very rich in steranes naphthalan).

	NAVS (N = 14)	Betamethasone (N = 13)
**Clinical type of RAU**	Minor RAU	Minor RAU
**Number of patients with 1 RAU lesion**	10	8
**Number of patients with 2 RAU lesions**	3	4
**Number of patients with 3 or more RAU lesions**	1	1
**Gender (M/F)**	5/9	8/5
**Age (years), mean ± SD**	52.35± 18.75	46 ± 17.24

#### Clinical improvement

*Primary outcome measures changes*. Reduction of number of lesions was evident only on 5^th^ day follow-up (Table 6), both in NAVS and in betamethasone group. The differences between the groups were not statistically significant (Table 7). Participants that received NAVS demonstrated a greater reduction of lesions diameter than participants who were treated with betamethasone but the differences between groups were not statistically significant (Table 6). Within-group analyses showed a significant reduction of lesions diameter both on 3^rd^ and on 5^th^ day follow-up for the NAVS group, while in betamethasone group a significant reduction of lesions diameter only on 5^th^ day the was found. The results of post hoc analyses showing differences from baseline to 3^rd^ and 5^th^ day follow-up are presented in Table 7.

**Table 6:** Means and SDs of outcome measures for each group at each time point (RAS patients).

		NAVS			Betamethasone			Partial eta-squared^ф^	
		**Baseline**	**3**^**rd**^ **day follow-up**	**5**^**th**^ **day follow-up**	**Baseline**	**3**^**rd**^ **day follow-up**	**5**^**th**^ **day follow-up**	**Baseline vs 3**^**rd**^ **day follow-up**	**Baseline vs 5**^**th**^ **day follow-up**
**Primary outcomes**	*No of lesions*	1.64 (1.59)	1.57 (1.65)	0.71 (1.07)	1.46 (0.66)	1.46 (0.66)	0.61 (0.65)	0.038	0.00002
	*lesion diameter*	5.58 (2.91)	3.63 (2.65)	1.25 (1.53)	5.62 (2.03)	4.46 (2.68)	1.42 (2.61)	0.057	0.001
		**Baseline**	**3**^**rd**^ **day follow-up**	**5**^**th**^ **day follow-up**	**Baseline**	**3**^**rd**^ **day follow-up**	**5**^**th**^ **day follow-up**	**Baseline vs 3**^**rd**^ **day follow-up**	**Baseline vs 5**^**th**^ **day follow-up**
	*pain intensity*	51.21 (20.06)	24.28 (15.64)	7.79 (8.60)	58.08 (17.21)	23.53 (13.11)	5.61 (11.25)	0.009	0.045
		**Baseline**	**8**^**th**^ **day follow-up**		**Baseline**	**8**^**th**^ **day follow-up**		**Baseline vs 8**^**th**^ **day follow-up**	
**Secondary outcome**	*OHIP score*	25.07 (11.19)	13.14 (6.98)		30.08 (11.89)	14.85 (7.8)		0.0001	

^ф^ Effect size for treatment differences between NAVS and Betamethasone group.

**Table 7:** Within groups ANOVA—Post-hoc pairwise comparisons (RAS patients).

			NAVS			Betamethasone		
			Mean differ.	95% CI	p ^ᵻ^	Mean differ.	95% CI	p ^ᵻ^
**Primary outcomes**	*No of lesions*	Baseline vs 3^rd^ day follow-up	0.071	-0.41–0.56	1	0.00	-0.52–0.52	1
		Baseline vs 5^th^ day follow-up	0.928	0.44–1.41	**0.001**	0.846	0.32–1.37	**0.01**
	*lesion diameter*	Baseline vs 3^rd^ day follow-up	1.94	0.56–3.32	**0.003**	1.16	-0.55–2.87	0.277
		Baseline vs 5^th^ day follow-up	4.32	2.95–5.71	**<0.0001**	4.20	2.49–5.91	**<0.0001**
	*pain intensity*	Baseline vs 3^rd^ day follow-up	26.92	14.75–39.10	**<0.0001**	34.53	24.11–44.96	**<0.0001**
		Baseline vs 5^th^ day follow-up	43.42	31.25–55.60	**<0.0001**	52.46	42.04–62.88	**<0.0001**
**Secondary outcome**	*OHIP score*	Baseline vs 8^th^ day follow-up	11.92	7.72–16.14	**<0.0001**	15.23	8.36–22.09	**0.0004**

CI: Confidence Interval;

^ᵻ^bolded p-values represent statistically significant differences.

**Fig 3:**
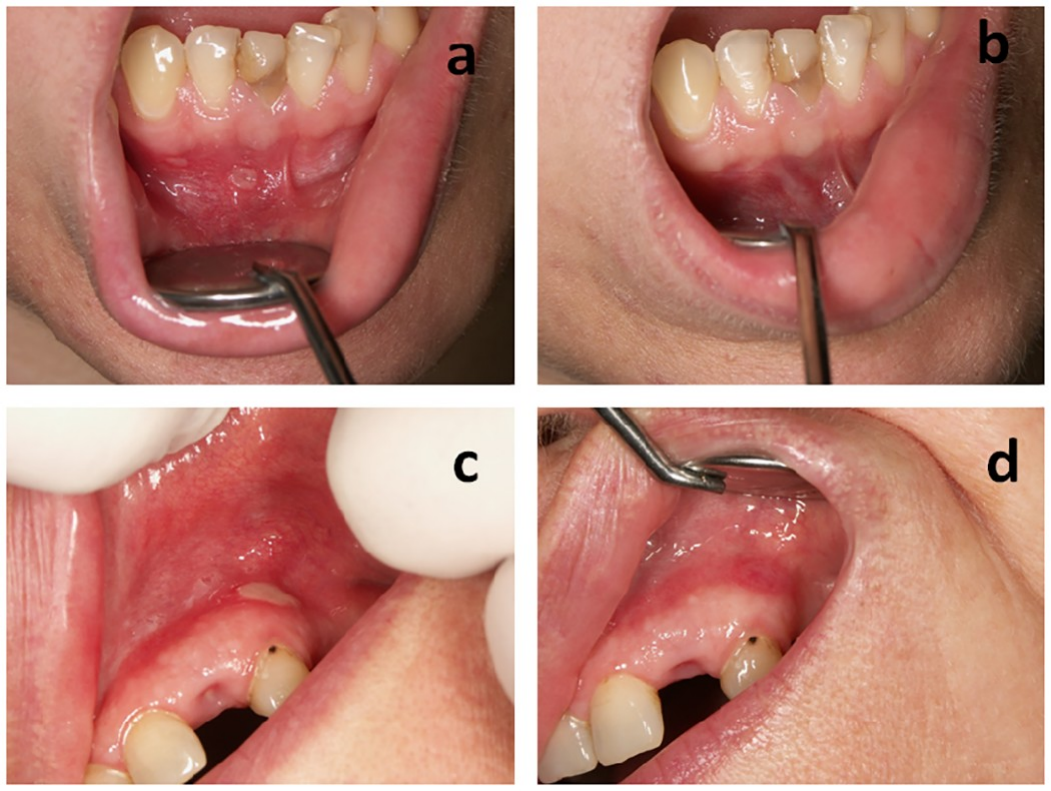
RAS lesion on day 0 (a) and on day 5 (b) of NAVS treatment, and on day 0 (c) and on day 5 (d) of betamethasone treatment.

Example of a patient with RAS lesion on day 0 and on day 5 of NAVS treatment is presented in Fig 3a and 3b, while the example of a patient with RAS lesion on day 0 and on day 5 of betamethasone treatment in Fig 3c and 3d.

Lesion size was expressed as the cumulative mean diameter of all ulcers in each group on day 0 and was expressed as 100%. There was no statistically significant difference between groups on days 3 and 5 in terms of lesion diameter reduction (day 3, *p* = 0.14; day 5 *p* = 0.79).

Regarding the changes in pain intensity, the within-group analyses showed a significant decrease in pain intensity on day 3 and day 5both for the NAVS and betamethasone group (Table 7) but the differences between groups were not statistically significant (Table 6).

When comparing changes in the reduction of VAS between groups, reduction in NAVS group compared to betamethasone both on day 3 (49.46%, 58.26, respectively) and day 5 (85.83%, 91.34%, respectively) was not statistically significant.

*Secondary outcome measures changes*. When examining within-group changes, improvement in OHIP-14 questionnaires at 8^th^ day follow-up was evident both in NAVS and in betamethasone group (Table 7) but the differences between groups did not reached statistical significance (Table 6).

When comparing changes in secondary outcome between NAVS and betamethasone, the relative decrease of OHIP-14 score was not significantly different (46.69, 47.88%, respectively; *p* = 0.88).

No significant differences between two tested groups were found when assessing the tolerability of the administered therapeutic agent, neither 30 minutes (t = 0.51; p = 0.61) nor 60 minutes (t = 0.46; p = 0.65) following the application.

## Discussion

Results of this study showed the efficacy of topical NAVS and betamethasone in the treatment of OLP and RAS lesions, as well as subjective symptoms reduction, but with no statistically significant differences. Further, NAVS didn’t showed any side effects. The gold standard for the treatment of immune-mediated oral diseases are topical steroids. The most commonly used steroids are betamethasone dipropionate, clobetasol propionate, triamcinolone acetonide, and fluocinonide [21–23]. Side-effects have been reported with long-term use of these agents. Hirsutism and moon face were reported in five patients with an erosive form of OLP treated with 0.05% clobetasol solution [24]. Dry mouth, bad taste, halitosis, lip swelling, and nausea have been reported in a randomized crossover study in which patients with symptomatic OLP were treated with fluticasone propionate and betamethasone solution for 6 weeks [19]. In our study, three out of fifteen (20%) OLP patients treated with betamethasone developed candidal infections. No cases of oral candidiasis were reported in patients with OLP treated with NAVS. No side effects in RAS patients were recorded. We demonstrated the efficacy of topical NAVS in the treatment of OLP lesions, shown by a significant reduction of hyperkeratosis, erythema and ulcerations (*p* = 0.0001). Although the overall clinical improvement in the NAVS group was slightly higher (667.55%), compared with 66.03% in the betamethasone group, the difference was not statistically significant (*p* = 0.935). After completion of the treatment, all patients had remission of the disease without the need for further therapy. There were no reported relapses during 4-week follow-up.

Currently, there is no widely accepted scoring system for severity of OLP lesions. There is a validated tool for assessing patient-related outcome measure (COMDQ) used for oral mucosal diseases. Unfortunately, at a time of designing the study protocol, COMDQ was not validated in Croatian. In our study, we had chosen a system [15] that is rather detailed and elaborated [25]. However, the authors highlight the lack of subjective assessment of pain and discomfort. Thus, we supplemented the clinical scoring system with a VAS scale and the OHIP 14 questionnaire to detect subjective discomfort and disease impact on everyday life. We found a reduction on OHIP 14 scores in the betamethasone group of 65.74%, while NAVS reduced OHIP 14 by 67.12%, a statistically insignificant difference (*p* = 0.81). Subjective symptoms, assessed by VAS scale, were also successfully reduced for both NAVS (96.241%), and betamethasone (93.24%). From a total of 27 patients with RAS, 14 were treated with NAVS and 13 with betamethasone. Reduction in the number of lesions was not noted on day 3 in either group. There was a marked reduction of number of lesions on day 5, with insignificantly less in the NAVS group compared with the betamethasone group. On days 3 and 5, reduction in the diameter of the lesions in the NAVS group (percentage of cumulative residual diameter 20.54%) was insignificantly better than that of the betamethasone group (percentage of cumulative residual diameter: 24.43%). Because the natural history of RAS is such that the number and diameter of lesions decrease over time, we need to compare the rates of change in order to objectively measure the effect of NAVS. We observed that RAS lesions treated with NAVS healed at a similar rate as those treated with betamethasone. Topical administration of NAVS in patients with RAS reduced subjective symptoms, as assessed by the OHIP 14 questionnaire and VAS. There were no statistically significant differences in the reduction of the OHIP-14 and VAS score between the groups.

There are several limitations to this study. First, NAVS cannot be properly defined as a standard pharmaceutical substance, as it is a natural geogenic product; NAVS has various constituents, of which some hydrocarbon fractions cannot be analyzed by existing methods (i.e., gas chromatography–mass spectrometry). Thus, we cannot discuss a concentration of NAVS. We know only that in our sample, steranes were present at concentration of around 18%. Second, subjects with the non-erosive form of OLP were included. Of a total of 30 patients, 21 patients had non-erosive OLP, and nine had erosive OLP. The erosive form causes the greatest intensity of pain and discomfort. The atrophic form, whose main clinical feature is atrophy and inflammation of the oral mucosa, also causes pain and discomfort, although of lower intensity. Published literature data also include patients with non-erosive OLP [26–30]. We included two oral conditions, as they both require similar treatment approaches. Third, three of OLP patients in the betamethasone group developed candida infection, which necessitated treatment with miconazole. These patients had total resolution of their infections by the end of 28 days. However, we do not know if miconazole enhances the healing of OLP lesions. Fourth, NAVS was prepared in an adhesive paste, which retains an otherwise oily substance longer at the site of the application. We cannot exclude the possible therapeutic effect of the paste itself, which could mechanically protect the lesion from external influences and thus favorably affect healing. Nevertheless, based on previous studies, we might conclude that the effect of adhesive paste on healing is minimal [28]. Finally, the sample size for this study, although based on power analysis, was still relatively small, which could potentially lead to a certain type of ‘bias’ and thus made the study exposed to imbalances between groups [31].

## Conclusions

To summarize, although NAVS and betamethasone are successful in treatment of OLP and RAS there was no evidence of differences between the two compared interventions. However, the results of this trial investigating the treatment efficacy of NAVS in comparison to betamethasone are promising because NAVS offerd stronger benefit particularly in RAS lesion diameter reduction after short period of time.

## Supporting information

S1 File Consort checklist. (DOC)

S2 File Clinical trial protocol Croatian version. (DOC)

S3 File Clinical trial protocol English version. (DOCX)

S4 File In vitro cytotoxicity study of NAVS_cro version. (PDF)

S5 File Preclinical toxicological study of NAVS_cro version. (PDF)

S6 File Determination of mutagenic or premutagenic potential of the tested sample of PY naphthalan_cro version. (PDF)

S7 File English version of the conclusions S5-7. (DOCX)

S8 File Clinical scoring of OLP and RAS patients_excel tbl. (XLSX)
