# Supplementary material for: Topical NAVS naphthalan for the treatment of oral lichen planus and recurrent aphthous stomatitis: A double blind, randomized, parallel group study
Source: PLoS One. 2021 Apr 8;16(4):e0249862. doi: 10.1371/journal.pone.0249862 (PMC8031371; doi:10.1371/journal.pone.0249862)
Supplement: S2 File — (DOC) [file pone.0249862.s002.doc]

Ana Andabak Rogulj,dr.stom. Etičko povjerenstvo

Stomatološki fakultet Stomatološki fakultet

Zavod za oralnu medicinu Petrinjska 34

Stomatološki fakultet 10000Zagreb

Gundulićeva 5

10000 Zagreb

Mentor: Doc.dr.sc. Ivan Alajbeg

Zavod za oralnu medicinu

Stomatološki fakultet

ETIČKI PROTOKOL

Predmet: Učinak topikalno primijenjenog nearomatskog naftalana (NAVS) u liječenju oralnog lihena planusa i rekurentnih aftoznih ulceracija

1.Svrha, ciljevi i hipoteza istraživanja

Svrha istraživanja je ispitati učinkovitost nearomatskog naftalana (NAVS) u liječenju oralnog lihena planusa i rekurentnih aftoznih ulceracija. Cilj istraživanja je uvesti novo ljekovito sredstvo, ukoliko objektivna klinička evaluacija nearomatskog naftalana pokaže djelotvornost u liječenju oralnih bolesti imunološke geneze.

2.Metodologija istraživanja

Istraživanje će se provesti na Zavodu za oralnu medicinu Stomatološkog fakulteta u Zagrebu na uzorku od ukupno 80 ispitanika (40 RAU, 40 OLP). Metodom slučajnog uzorka biti će podijeljeni u dvije skupine: ispitnu i kontrolnu. Aplikacija NAVS-a predviđena je u obliku „orabaze“ (paste koja adherira na oralnu sluznicu) kod ispitanika u akutnoj fazi bolesti. Ispitanicima kontrolne skupine umjesto nearomatskog naftalana primijenit će se današnji zlatni standard - topikalno primijenjen kortikosteroid u „orabazi“.

3.Sudionici istraživanja(ispitanici)

Sudionici istraživanja su pacijenti Zavoda za oralnu medicinu Stomatološkog fakulteta u Zagrebu, s potvrđenom dijagnozom 1) oralnog lihena planusa (klinička dijagnoza i histopatološki nalaz prema Svjetskoj zdravstvenoj organizaciji) (1) ili 2) s rekurentim aftoznim stomatitisom (prema Lehneru; 2 + epizode godišnje) (2).

4.Izabiranje ispitanika

Punoljetnim bolesnicima koji redovito dolaze radi liječenje na Zavod, zadovoljavaju uključne kriterije, a nemaju niti jedan od isključnih kriterija, bit će ponuđeno sudjelovanje u istraživanju. Sudjelovanje će biti na dobrovoljnoj bazi. Svi će biti detaljno upućeni u dizajn studije i za sudjelovanje će trebati potpisati informirani pristanak.

Uključni kriteriji:

jedna od dviju navedenih dijagnoza

Isključni kriteriji:

manje od 18 godina, hematološki deficiti, trudnoća, upalne crijevne bolesti, značajne imunološke deficijencije, trenutna konkomitantna sistemska ili lokalna protuupalna terapija (kortikosteroidi, nesteroidni antireumatici,...) (3-5).

5.Rizici i korist

Rizici

NAVS je siguran za uporabu na ljudima. NAVS je vrlo opsežno istraživan *In vitro* i *In vivo*, na životinjskim modelima i na ljudima (6-10). Studije genotoksičnosti, mutageničnosti, mikrobiološke ispravnosti, sadržaja teških metala i nemetala i iritabilnosti pokazuju njegovu potpuno sigurnu primjenu, neovisno o dozi (11). Dosadašnja klinička istraživanja na dermatološkim bolesnicima pokazuju poželjan terapijski učinak, bez lokalnih i sistemskih nuspojava(12, 13). Ishodišna sirovina za NAVS (njegov nepurificirani prekursor) je obični naftalan, već desetljećima u uporabi kao kupka za cijelo tijelo u Specijalnoj bolnici „Naftalan“, Ivanić Grad, bez opisanih nuspojava niti odraza na biokemijske ili hematološke parametre (14).

Novi dokazi neštetnosti NAVS-a:

1) NAVS ne pokazuje citotoksičnost na kulturama humanih keratinocita ni fibroblasta. Molim obratiti pozornost na podatke istraživanja Farmaceutsko biokemijskog fakulteta o citotoksičnosti NAVS-a na staničnim kulturama (u privitku, strana 16, označeno žutim).

2) Također, studije toksičnosti „In vivo“ na laboratorijskim životinjama (miševi C57B1) kojima je davan NAVS 7 dana sondom „per os“ pokazuju da nije došlo ni do kakvih promjena biokemijskih i hematoloških parametara (studija načinjena na IRB-u, u privitku, molim pogledati str. 10, označeno žutim).

3) „Amesov“ test mutageničnosti napravljen na manje purificiranom naftalanu („PY“ naftalanu koji još sadrži minimalnu količinu aromata, a kojem je NAVS superioran) pokazuje nepostojanje mutageničnog potencijala (istraživanje napravljeno na IRB-u, molim vidjeti u privitku, str. 6, označeno žutim). Molimo uzeti u obzir da su priloženi podaci tajni prije javne objave.

U smislu nuspojava u aktivnoj skupini rizika u ovom istraživanju za ispitanike nema.

Rizik u smislu odgađanja sanacije oralne bolesti u ispitnoj skupini je moguć ukoliko ispitivano sredstvo nije djelotvorno. Kako je u kontrolnoj skupini riječ o terapijskom „zlatnom standardu“ kojim se danas redovito liječe bolesnici s navedenim dijagnozama, rizik je identičan uobičajenom terapijskom riziku. Tu je riječ o sredstvu poznate djelotvornosti, s kojim se novo sredstvo uspoređuje. Rizici kratkotrajne uporabe topikalnih kortikosteroida su klinički beznačajni, dok njihova dugotrajna uporaba nije preporučljiva zbog nuspojava (atrofija sluznice, sekundarna infekcija, sistemska apsorpcija i supresija nadbubrežne žlijezde) (3). Upravo zbog nuspojava standardne terapije, ovim se istraživanjem traži alternativa kortikosteroidima. Mjera učinkovitosti NAVS-a bit će učinak što sličniji „zlatnom standardu“.

Koristi

Pacijenti će proći kroz vrlo blisko nadzirani terapijski protokol u kojem će primati ili standarnu terapiju ili ljekovito sredstvo bez kortikosteroida, koji danas predstavljaju terapiju izbora navedenih bolesti. To će sredstvo dobiti na licu mjesta, besplatno.

6.Privatnost i povjerljivost

Podaci su anonimni. Identitet će biti zaštićen na način da će identifikacijski broj pacijenta odgovarati identifikacijskom broju na anketnom upitniku. Podaci će biti korišteni samo u svrhu ovog istraživanja.

7.Kompenzacija

U ovom istraživanju nije predviđena kompenzacija.

8.Sukob interesa

Ne postoji moguć sukob interesa. Interes je znanstveni, a sudionici istraživanja rade na projektu Ministarstva znanosti, obrazovanja i športa RH „Nearomatski naftalan – istraživanje sastava i bioloških učinaka na epitelna tkiva“, broj 065-0650445-1277.

9.Postupak pribavljanja informiranog pristanka

S obzirom da je riječ o akutnim ili subakutnim stanjima, pacijentu se brzo treba ponuditi terapijsko rješenje za njegovu bolest. Informacija za ispitanike daje se pacijentima tijekom pregleda. Verbalno im se objasne detalji i odgovori se na sva pitanja. Ukoliko pacijentu treba više vremena za odluku, tada ga se ne uključuje, već mu se ponudi mogućnost uključivanja tijekom neke buduće reaktivacije bolesti. Pacijenti koji potpišu informirani pristanak uključuju se odmah u protokol.

10.Uporaba bioloških uzoraka i stomatološke/medicinske dokumentacije?

Podaci će se koristiti samo za potrebe istraživanja.

11.Sekundarna uporaba bioloških uzoraka i stomatološke/medicinske dokumentacije.

N/A

12.Dodatne etičke ocjene

Nema ih.

13.Klinička istraživanja

1)Klinički protokol

2)Primjerak informacije za ispitanike

3)Primjerak informiranog pristanka

4)CV istraživača i potvrda predstojnika Zavoda

Istraživač: Mentor:

Ana Andabak Rogulj,dr.stom. Doc.dr.sc. Ivan Alajbeg

(potpis) (potpis)

Zagreb, 12. studenog 2010.

Literatura

1. WHO Collaborating Centre for Oral Precancerous Lesions. Definition of leukoplakia and related lesions: an aid to studies on oral precancer. Oral Surg Oral Med Oral Pathol 1978; 46: 518-39.
2. Lehner T. Autoimmunity in oral disease with special referrence to recurrent oral ulcerations. Proc R Soc Med 1968; 61: 515-24.
3. Lo Muzio L, della Valle A, Mignona MD et al. The treatment of oral aphthous ulceration or erosive lichen planus with topical clobetasol propionate in three preparations: a clinical and pilot study on 54 patients. J Oral Pathol Med 2001; 30: 611-7.
4. Rodriguez M, Rubio JA, Sanchez R. Effectiveness of two oral pastes for the treatment of recurrent aphthous stomatitis. Oral Diseases 2007; 13: 490-4.
5. Nolan A, Baillie C, Badminton J et al. The efficacy of topical hyaluronic acid in the management of recurrent aphthous ulceration. J Oral Pathol Med 2006; 35: 461-5.
6. Alajbeg I, Ivankovic S, Alajbeg, IZ et al. Antiproliferative effect of non-aromatic oil fractions on squamous cell carcinoma VII: in vitro and preliminary in vivo results. Period Biol 2002; 104: 89-94.
7. Li MW, Lin RZ, Liao YS et al. Organic geochemistry of oils and condensates in the Kekeya Field, Southwest Depression of the Tarim Basin (China). Org Geochem 1999; 30: 15-37.
8. Alajbeg I, Ivankovic S, Jurin M et al. : Non-aromatic naphthalane as a potential healing medium. Period Biol 2002; 104: 81-87.
9. Stoilov I, Smith SL, Watt DS et al. Synthesis of biological markers in fossil fuels. H-1 and C-13 NMR analysis of C-23 and C-24 diastereomers of 5-alpha-dinosterane. Magn Reson Chem 1994; 32: 101-6.
10. Thaci D, Schindewolf M, Smeh-Skrbin A et al. Heavy naphthen oil exhibits antipsoriaticefficacy in vivo and antiproliferative as well as differentation-including effects on keratinocytes in vitro. Arch Dermatol 2000; 136: 678-9.
11. Alajbeg I, Dinter G, Alajbeg A et al. Study of Croatian non-aromatic naphthalane constituents with skeletons analogous to bioactive compounds. J Chromatogr A 2001; 918: 127-34.
12. Alajbeg I, Krnjević-Pezić G, Smeh-Skrbin A et al. Non-aromatic naphthalane preparation; preliminary clinical study in the treatment of psoriasis vulgaris. J Pharm Biomed Anal 2001; 26: 801-9.
13. Smeh-Skrbin A, Dobrić I, Krnjević-Pezić G et al. Naphthalan in the treatment of patients with atopic dermatitis. Acta Dermatovenerol Croat 2007; 15: 15-9.
14. Krnjević-Pezić G, Vržogić P, Ostrogović Ž et al. Some hematological and biochemical parametrers in psoriatic patients treated with naphthalan. Acta Dermatovenerol Croat 1997; 5: 49-53.

**Klinički protokol**

**Učinak topikalno primijenjenog nearomatskog naftalana u liječenju oralnog lihena planusa i rekurentnih aftoznih ulceracija**

Svrha istraživanja je ispitati učinkovitost i sigurnost nearomatskog naftalana (NAVS) u liječenju oralnog lihena planusa i rekurentnih aftoznih ulceracija. Cilj istraživanja je uvesti novo ljekovito sredstvo, ukoliko objektivna klinička evaluacija nearomatskog naftalana

pokaže djelotvornost u liječenju oralnih bolesti imunološke geneze.

**Ispitanici i postupci**
Istraživanje će se provesti na Zavodu za oralnu medicinu Stomatološkog fakulteta u Zagrebu na uzorku od ukupno 80 ispitanika (40 RAU, 40 OLP). Metodom slučajnog uzorka bit će podijeljeni u dvije skupine: ispitnu i kontrolnu. Aplikacija NAVS-a predviđena je u obliku „orabaze“ (paste koja adherira na oralnu sluznicu) kod ispitanika u akutnoj fazi bolesti. Ispitanicima kontrolne skupine umjesto nearomatskog naftalana primijenit će se današnji zlatni standard - topikalno primijenjen kortikosteroid u „orabazi“.

Pokus će biti dvostruko slijepi. Jedan član tima, koji neće procjenjivati terapijski učinak, vodit će brigu o alokaciji ispitnih i kontrolnih pripravaka. Na kraju israživanja šifru ćemo otvoriti i napraviti statističku obradu podataka.

**RAU-rekurentne aftozne ulceracije**
U istraživanje će biti uključeno 40 odraslih pacijenata s rekurentnim aftoznim ulceracijama koje se pojavljuju najmanje dva puta godišnje i koji imaju najmanje jednu ulceraciju prilikom aplikacije NAVS-a/ u usnoj šupljini. Svi pacijenti biti će podvrgnuti hematološkim pretragama prije uključivanja u studiju. Hematološke pretrage uključuju kompletnu krvnu sliku (KKS), željezo (Fe), B12 i antiendomizijska protutijela. Iz studije će biti isključeni pacijenti koji imaju poremećene hematološke nalaze, koji uzimaju steroidne i nesteroidne protuupalne lijekove (NSAID) ili kemoterapijske lijekove, te pacijenti s mogućom preosjetljivošću na zubne paste i vodice za ispiranje usne šupljine (anamnestička procjena) (1).
Simptomatologija i klinička slika:

Procjenjivat će se broj i veličina lezija na dan 0, 2 i 5 (2) . Bilježit će se duljina postojanja simptoma u danima.

Pri prvom posjetu prije i nakon aplikacije NAVS-a / kontrole pacijenti će na vizualno-analognoj skali (VAS) od 10 cm odrediti stupanj bolnosti i nelagode. Pacijenti će dobiti dovoljno sredstva za aplikaciju 3x dnevno tijekom 7 dana. Tijekom 7 dana pacijenti će voditi dnevnik simptomatologije i broja ulceracija. Stupanj bolnosti i nelagode procijenit će se dodatno 30 i 60 minuta nakon aplikacije sredstva, kod kuće, i uvesti u dnevnik. Kontrola pacijenata će se vršiti na dane 0, 2 i 5. Osmog dana pacijenti će na pregled donijeti dnevnike i ocijeniti primijenjenu terapiju (1).
Klinički nalaz će biti fotografiran prilikom pregleda.

**Oralni lihen planus**
U istraživanje će biti uključeno 40 odraslih pacijenata sa klinički evidentnim oralnim lihenom. Histopatološka potvrda primijeniti će se isključivo u klinički sumnjivim slučajevima (3). Iz istraživanja će biti isključeni pacijenti s bolestima hepatobilijarnog sustava ili s lihenoidnom reakcijom (amalgam, lijekovi) (4).
Usna šupljina svakog pacijenta podijelit će se u 10 regija (tablica). Težina pojedine lezije u regijama, svaki tjedan, bodovat će se prema prisutnosti hiperkeratoze, erozije/eritema i/ili ulceracije prema slijedećem:

1. Hiperkeratoza    0-1   (0=bez hiperkeratotičnih strija, 1=prisutnost hiperkeratotičnih   strija ili papula)
2. Erozija/eritem    0-3   (0=bez lezija, 1=lezije manje od 1 cm², 2=lezije veličine 1-3 cm², 3=lezije veće od 3 cm²)
3. Ulceracije           0-3   (0=bez lezija, 1=lezije manje od 1 cm², 2=lezije veličine 1-3 cm², 3=lezije veće od 3 cm²).

Za svaki od 3 promatrana klinička parametra vrijednosti su dobivene sumom bodova svih deset regija: hiperkeratoza ∑R, eritem ∑E, ulceracija ∑U (REU)→∑R+∑ (EX1.5)+∑(UX2.0).
Fotografije zahvaćenih područja usne šupljine napravit će se prilikom prvog posjeta, te 4. i 8. tjedna nakon započete terapije. Tri kalibrirana ispitivača neovisno će pregledati i ocijeniti svaku fotografiju pojedinog pacijenta. Druga evaluacija fotografija biti će tjedan dana nakon prvog posjeta kako bi se procijenila objektivnost očitanih vrijednosti prilikom prvog posjeta. Nakon što kalibrirani ispitivači pregledaju fotografije dva puta u razmaku od tjedan dana, dobiveni rezultati analizirati će se pomoću Spearmanove „rank“ korelacije kako bi se utvrdila „inter-observer“ i „intra-observer“ pouzdanost (5).

| ZAHVAĆENA POVRŠINA | HIPERKERATOZA | ERITEM | ULCERACIJA |
| --- | --- | --- | --- |
| Gornja/donja labijalna mukoza | 0   1 | 0  1  2  3 | 0  1  2  3 |
| Bukalna mukoza (desno) | 0   1 | 0  1  2  3 | 0  1  2  3 |
| Bukalna mukoza (lijevo) | 0   1 | 0  1  2  3 | 0  1  2  3 |
| Dorzum jezika | 0   1 | 0  1  2  3 | 0  1  2  3 |
| Ventrum jezika | 0   1 | 0  1  2  3 | 0  1  2  3 |
| Dno usne šupljine | 0   1 | 0  1  2  3 | 0  1  2  3 |
| Tvrdo nepce | 0   1 | 0  1  2  3 | 0  1  2  3 |
| Meko nepce | 0   1 | 0  1  2  3 | 0  1  2  3 |
| Maksilarna gingiva | 0   1 | 0  1  2  3 | 0  1  2  3 |
| Mandibularna gingiva | 0   1 | 0  1  2  3 | 0  1  2  3 |
| UKUPNO |  |  |  |

Pibooniyom et al. 2005.

**Za obje skupine bolesnika**

Prije i tijekom same terapije intenzitet boli i nelagode odredit će se pomoću vizualno analogne ljestvice te pomoću OHIP-a 14 (za RAU i OLP) po završetku terapije za obje skupine.
Vrijeme bez uzimanja sredstva („wash-out period“) predviđeno je u trajanju od 2 tjedna , tijekom kojeg pacijenti ne smiju uzimati ništa što bi moglo mijenjati kliničku sliku bolesti (sistemski kortikosteroidi, antimikotici, imunosupresivi).

**Statistička obrada**

Nakon završetka istraživanja otvorit će se randomizacijski kod. Wilcoxonovim testom za parne uzorke će se ocijeniti učinak terapije u svakoj od skupina. Između skupina učinak će biti procijenjen Mann-Whitneyevim testom.

Ispitanici će kroz 14 pitanja ocijeniti koliko često su imali problema sa ustima u zadnjih mjesec dana na skali (Likertova skala) od 0-4 (0-nikad, 1-gotovo nikad, 2-ponekad, 3-često, 4-uvijek) (6).

OHIP-CRO14:

1. Jeste li imali ili imate **poteškoća pri izgovaranju riječi** zbog problema sa ustima?
2. Jeste li osjetili ili **osjećate neugodan okus** zbog problema sa ustima?
3. Jeste li imali ili imate **jake bolove u ustima**?
4. Je li vam bilo ili vam je **nelagodno jesti pojedinu vrstu hrane** zbog problema sa ustima?
5. Jeste li **razmišljali** o vašim ustima?
6. **Osjećate li tjeskobu** zbog problema sa ustima?
7. Smatrate li da vam je **prehrana nezadovoljavajuća** zbog problema sa ustima?
8. Jeste li morali **prekidati obrok** zbog problema sa ustima?
9. Je li vam **se teže opustiti** zbog problema sa ustima?
10. Jeste li se **osjetili imalo neugodno** zbog problema sa ustima?
11. Jeste li bili **razdražljivi prema drugima** zbog problema sa ustima?
12. Jeste li imali **problema u obavljanju svakodnevnih poslova** zbog problema sa ustima?
13. Smatrate li da vam **život pruža manje zadovoljstva** zbog problema sa ustima?
14. Je li vam se dogodilo da **uopće ne funkcionirate** zbog problema sa ustima?
15. Nolan A, Baillie C, Badminton J et al. The efficacy of topical hyaluronic acid in the management of recurrent aphthous ulceration. J Oral Pathol Med 2006; 35: 461-5.
16. Khandwala A, Van Inwegen RG, Alfano MC. 5% amlexanox oral paste, a new treatment for recurrent minor aphthous ulcers: I. Clinical demonstration of acceleration of healing and resolution of pain. Oral Surg Oral Med Oral Pathol Oral Radiol Endod 1997; 83: 222-30.
17. Tyldesley WR, Harding SM. Betamethasone valerate aerosol in the treatment of oral lichen planus. Br J Dermatol 1997; 96: 659-62.
18. Zakrzewska JM, Chan ES-Y, Tornhill MH. A systematic review of placebo-controlled randomized clinical trials of treatments used in oral lichen planus. Br J Dermatol 2005; 153: 336-41.
19. Piboonniyom SO, Treister N, Pitiphat W et al. Scoring system for monitoring oral lichenoid lesions: A preliminary study. Oral Surg Oral Med Oral Pathol Oral Radiol Endod 2005; 99: 696-703.
20. Petricević N, Celebić A, Papić M et al. The Croatian version of the Oral Health Impact Profile Questionnaire. Coll Antropol 2009; 33: 841-7.
