# Supplementary material for: Topical NAVS naphthalan for the treatment of oral lichen planus and recurrent aphthous stomatitis: A double blind, randomized, parallel group study
Source: PLoS One. 2021 Apr 8;16(4):e0249862. doi: 10.1371/journal.pone.0249862 (PMC8031371; doi:10.1371/journal.pone.0249862)
Supplement: S3 File — (DOCX) [file pone.0249862.s003.docx]

Ana Andabak Rogulj, DMD. Ethics Committee

School of Dental medicine School of Dental medicine

Department of Oral medicine Petrinjska 34

University of Zagreb 10000 Zagreb

Gundulićeva 5

10000 Zagreb

Mentor: Assistant professor Ivan Alajbeg

Department of Oral medicine

School of Dental medicine

**ETHICAL PROTOCOL**

Subject: Topical “Non-aromatic very rich in steranes” (NAVS) naphthalan for the treatment of oral lichen planus and recurrent aphthous stomatitis

1. Purpose, goals and hypothesis of the research

The purpose of the study was to examine the efficacy of non-aromatic naphthalan (NAVS) in the treatment of oral lichen planus and recurrent aphthous ulcers. The aim of the study is to introduce a new medication, should the objective clinical evaluation of non-aromatic naphthalan show efficacy in the treatment of oral immune mediated diseases.

2. Research methodology

The research will be conducted at the Department of Oral Medicine, School of Dental medicine in Zagreb on a sample of up to 80 subjects (40 RAU, 40 OLP). Subjects will be randomized into two groups: test and control. NAVS application will be in the form of "orabase" (a paste that adheres to the oral mucosa) in subjects in the acute phase of the disease. Today's gold standard - topically applied corticosteroids in orabase will be applied to the subjects of the control group.

3. Research participants

Participants in the study are patients of the Department of Oral Medicine, School of Dental medicine University of Zagreb, with a confirmed diagnosis of 1) oral lichen planus (clinical diagnosis and histopathological findings according to the World Health Organization) (1) or 2) with recurrent aphthous stomatitis (according to Lehner; 2 + episodes per year ) (2).

4. Selection of participants

Adult patients who regularly come to the Department for treatment, who meet the inclusion criteria, and do not have any of the exclusion criteria, will be offered to participate in the research. Participation will be on a voluntary basis. All patients will be instructed in detail with study design and will need to sign an informed consent form to participate.

Inclusion criteria:

One of the two diagnoses listed.

Exclusion criteria:

Less than 18 years, hematological deficits, pregnancy, inflammatory bowel disease, significant immune deficiencies, current concomitant systemic or local anti-inflammatory therapy (corticosteroids, NSAID, ...) (3-5).

5. Risks and benefits

Risks

NAVS is safe for use on humans. NAVS has been extensively studied in vitro and in vivo, in animal models and in humans (6-10). Studies of genotoxicity, mutagenicity, microbiological safety, heavy metal and non-metal content and irritability showed that application was completely safe, regardless of dose (11). Previous clinical studies in dermatological patients have shown a favourable therapeutic effect, without local and systemic side effects (12, 13). The starting material for NAVS (its non-purified precursor) is ordinary naphthalan, which has been used for decades as a whole body bath at the Special Hospital "Naftalan", Ivanić Grad, without side effects or impact on biochemical or hematological parameters (14).

New evidence of NAVS safety:

1) NAVS does not show cytotoxicity on human keratinocyte or fibroblast cultures. Please pay attention to the research data of the Faculty of Pharmacy and Biochemistry on the cytotoxicity of NAVS on cell cultures (attached, page 16, marked in yellow).

2) Also, “In vivo” toxicity studies in laboratory animals (C57B1 mice) in which NAVS was given for 7 days with a peroral probe showed that no changes in biochemical and hematological parameters were occurred (Rudjer Bošković Institute study, attached). , please see page 10, marked in yellow).

3) "Ames" mutagenicity test performed on less purified naphthalan ("PY" naphthalan which still contains a minimum amount of aromatics, and to which NAVS is a superior product) didn’t show mutagenic potential (research done at the Rudjer Bošković Institute, please see attached, p. 6 , marked in yellow). Please note that the attached information is confidential prior to public disclosure.

Three above mentioned documents with expert analysis are attached (pdf). As described above, the most important findings are highlighted yellow. English translation of the findings highlighted in yellow are provided in a separate word document.

In terms of side effects, there are no perceived risk for the subjects in the active group in this study. The risk in terms of delaying the remediation of oral disease in the test group is possible if the tested agent is not effective. As the control group is the therapeutic "gold standard" which is now used to regularly treat patients with these diagnoses, the risk is identical to the usual therapeutic risk. It is an agent of known efficacy, with which the new one is compared. The risks of short-term use of topical corticosteroids are clinically insignificant, while their long-term use is not recommended due to side effects (mucosal atrophy, secondary infection, systemic absorption, and adrenal suppression) (3). Just because of the side effects of standard therapy, this research is looking for an alternative to corticosteroids. The measure of the effectiveness of NAVS will be the effect as similar as possible to the "gold standard".

Benefits

Patients will undergo through a very closely monitored therapeutic protocol in which they will receive either standard therapy or a drug without corticosteroids, which today represent the therapy of choice for these diseases. They will receive this agents free of charge.

6. Privacy and confidentiality

The data are anonymous. The identity will be protected in such a way that the patient identification number will correspond to the identification number on the survey questionnaire. The data will be used for the purpose of this research only.

7. Compensation

No compensation is anticipated in this study.

8. Conflict of interest

There is no possible conflict of interest. The interest is scientific, and the research participants are working on the project of the Ministry of Science, Education and Sports of the Republic of Croatia "Non-aromatic naphthalan - research of composition and biological effects on epithelial tissues", number 065-0650445-1277.

9. Procedure for obtaining informed consent

Since these are acute or subacute conditions, a therapetuic solution for the disease should be offered as soon as possible. Information is given to patients during the examination. The details are verbally explained to them and all questions are answered. If patients need more time to make a decision, then they are not included, but are offered the opportunity to be included during some future reactivation of the disease. Patients who sign informed consent are immediately included in the protocol.

10. Use of biological samples and dental / medical documentation?

The data will only be used for research purposes.

11. Secondary use of biological samples and dental / medical documentation.

N/A.

12. Additional ethical assessments

None.

13. Clinical research

1) Clinical protocol

2) A copy of the information for the respondents

3) A copy of the informed consent

4) CV of the researcher and certificate of the head of the Department of Oral medicine.

  Investigator: Mentor:

Ana Andabak Rogulj, DMD. Assistant professor Ivan Alajbeg (signature) (signature)

Zagreb, November 12, 2010.

Literature:

1. WHO Collaborating Centre for Oral Precancerous Lesions. Definition of leukoplakia and related lesions: an aid to studies on oral precancer. Oral Surg Oral Med Oral Pathol 1978; 46: 518-39.
2. Lehner T. Autoimmunity in oral disease with special referrence to recurrent oral ulcerations. Proc R Soc Med 1968; 61: 515-24.
3. Lo Muzio L, della Valle A, Mignona MD et al. The treatment of oral aphthous ulceration or erosive lichen planus with topical clobetasol propionate in three preparations: a clinical and pilot study on 54 patients. J Oral Pathol Med 2001; 30: 611-7.
4. Rodriguez M, Rubio JA, Sanchez R. Effectiveness of two oral pastes for the treatment of recurrent aphthous stomatitis. Oral Diseases 2007; 13: 490-4.
5. Nolan A, Baillie C, Badminton J et al. The efficacy of topical hyaluronic acid in the management of recurrent aphthous ulceration. J Oral Pathol Med 2006; 35: 461-5.
6. Alajbeg I, Ivankovic S, Alajbeg, IZ et al. Antiproliferative effect of non-aromatic oil fractions on squamous cell carcinoma VII: in vitro and preliminary in vivo results. Period Biol 2002; 104: 89-94.
7. Li MW, Lin RZ, Liao YS et al. Organic geochemistry of oils and condensates in the Kekeya Field, Southwest Depression of the Tarim Basin (China). Org Geochem 1999; 30: 15-37.
8. Alajbeg I, Ivankovic S, Jurin M et al. : Non-aromatic naphthalane as a potential healing medium. Period Biol 2002; 104: 81-87.
9. Stoilov I, Smith SL, Watt DS et al. Synthesis of biological markers in fossil fuels. H-1 and C-13 NMR analysis of C-23 and C-24 diastereomers of 5-alpha-dinosterane. Magn Reson Chem 1994; 32: 101-6.
10. Thaci D, Schindewolf M, Smeh-Skrbin A et al. Heavy naphthen oil exhibits antipsoriaticefficacy in vivo and antiproliferative as well as differentation-including effects on keratinocytes in vitro. Arch Dermatol 2000; 136: 678-9.
11. Alajbeg I, Dinter G, Alajbeg A et al. Study of Croatian non-aromatic naphthalane constituents with skeletons analogous to bioactive compounds. J Chromatogr A 2001; 918: 127-34.
12. Alajbeg I, Krnjević-Pezić G, Smeh-Skrbin A et al. Non-aromatic naphthalane preparation; preliminary clinical study in the treatment of psoriasis vulgaris. J Pharm Biomed Anal 2001; 26: 801-9.
13. Smeh-Skrbin A, Dobrić I, Krnjević-Pezić G et al. Naphthalan in the treatment of patients with atopic dermatitis. Acta Dermatovenerol Croat 2007; 15: 15-9.
14. Krnjević-Pezić G, Vržogić P, Ostrogović Ž et al. Some hematological and biochemical parametrers in psoriatic patients treated with naphthalan. Acta Dermatovenerol Croat 1997; 5: 49-53.

**Clinical Protocol**

**Effect of topical “Non-aromatic very rich in steranes” (NAVS) naphthalan for the treatment of oral lichen planus and recurrent aphthous stomatitis**

The purpose of this study was to evaluate efficacy and safety of non-aromatic naphthalan (NAVS) in the treatment of oral lichen planus and recurrent aphthous ulcers. The aim of the study is to introduce a new drug, if an objective clinical evaluation of NAVS shows efficacy in the treatment of oral immune mediated diseases.

**Materials and Methods**

The research will be conducted at the Department of Oral Medicine, School of Dental Medicine University of Zagreb on a sample up to 80 subjects (40 RAU, 40 OLP). Subjects will be divided by the method of random sampling into two groups: test and control. NAVS application will be in the form of "orabase" (a paste that adheres to the oral mucosa) in subjects in the acute phase of the disease. Today's gold standard - topically applied corticosteroids in orabase will be applied to the subjects of the control group. The experiment will be double-blind. One member of the team, who will not evaluate the therapeutic effect, will take care of the allocation of test and control preparations. At the end of the study, we will open the code and perform statistical data analysis.

**RAU-recurrent aphthous ulcerations**

The study will include up to 40 adult patients with recurrent aphthous ulcers with at least to episode per year and who have at least one ulceration when applying NAVS / in the oral cavity. Prior to study enrollment, all patients will undergo hematological examinations. Haematological tests include complete blood count (CBC), iron (Fe), B12, and antiendomysial antibodies. Patients with impaired haematological findings, taking steroidal and non-steroidal anti-inflammatory drugs (NSAIDs) or chemotherapeutic drugs, and patients with possible hypersensitivity to toothpastes and mouthwashes (anamnestic assessment) will be excluded from the study (1).

Symptomatology and clinical features:

The number and size of the lesions on days 0, 2, and 5 will be assessed (2). The length of symptoms existence in days will be recorded. At the first visit, before and after the application of NAVS / control, patients will determine the degree of pain and discomfort on a visual-analog scale (VAS) of 10 cm. Patients will receive enough resources for application 3 times a day for 7 days. During the seven days, patients will record symptoms in pain diary and number of ulcerations. The degree of pain and discomfort will be assessed additionally 30 and 60 minutes after application of the agent, at home, and entered in the diary. Patient monitoring will be performed on days 0, 2 and 5. On the eighth day, patients will bring pain diaries for examination and evaluate the applied therapy (1).

The clinical finding will be photographed during the examination.

**Oral lichen planus**

The study will include 40 adult patients with clinically evident oral lichen. Histopathological confirmation will be used only in clinically suspicious cases (3). Patients with hepatobiliary system diseases or with a lichenoid reaction (amalgam, drugs) will be excluded from the study (4).

The oral cavity of each patient will be divided into 10 sites (Table). The severity of the lesions in each site will be scored every week according to the presence of reticular/hyperkeratotic, erosive/erythematous, and/or ulcerativelesion(s) as follows:

1. Reticular/hyperkeratotic lesions score from 0 to 1 (0 = no white striations, 1 = presence of white striations or keratotic papules);
2. Erosive/erythematous areas score from 0 to 3 by area of involvement (0 = no lesion, 1 = lesions less than 1cm^2^, 2 = lesions from 1 to 3 cm^2^, 3 = lesions greater than 3 cm^2^);
3. Ulcerative areas score from 0 to 3 by area of involvement (0 = no lesion, 1 = lesions less than 1cm^2^, 2 = lesions from 1 to 3 cm^2^, 3 = lesions greater than 3 cm^2^).

For each of the 3 clinical signs, a score will be derived by summation of the scores of all 10 areas: reticular score ∑R, erythema score ∑E, ulcerative score ∑U (REU score) → ∑R+∑(EX1.5)+∑(UX2.0).

Photographs of the affected areas of the oral cavity will be taken during the first visit, and at 4^th^ and 8^th^ weeks after the start of therapy. Three calibrated examiners will independently review and evaluate each photograph of each patient. A second evaluation of the photographs will be one week after the first visit to assess the objectivity of the values read at the first visit. After the calibrated examiners review the photographs twice at one-week intervals, the obtained results will be analyzed using Spearman’s “rank” correlation to determine “inter-observer” and “intra-observer” reliability (5).

| SITE | RETICULAR AREA | ERYTEMATHOUS AREA | ULCERATIVE AREA |
| --- | --- | --- | --- |
| Upper/lower labial mucosa | 0   1 | 0  1  2  3 | 0  1  2  3 |
| Right buccal mucosa | 0   1 | 0  1  2  3 | 0  1  2  3 |
| Left buccal mucosa | 0   1 | 0  1  2  3 | 0  1  2  3 |
| Dorsal tongue | 0   1 | 0  1  2  3 | 0  1  2  3 |
| Ventral tongue | 0   1 | 0  1  2  3 | 0  1  2  3 |
| Floor of mouth | 0   1 | 0  1  2  3 | 0  1  2  3 |
| Hard palate mucosa | 0   1 | 0  1  2  3 | 0  1  2  3 |
| Soft palate | 0   1 | 0  1  2  3 | 0  1  2  3 |
| Maxillary gingiva | 0   1 | 0  1  2  3 | 0  1  2  3 |
| Mandibular gingiva | 0   1 | 0  1  2  3 | 0  1  2  3 |
| TOTAL |  |  |  |

Pibooniyom et al. 2005.

**For both groups of patients**

Before and during therapy, the intensity of pain and discomfort will be determined using a visual analogue scale and OHIP 14 questionnaire (for RAU and OLP) at the end of therapy for both groups. A wash-out period is provided for 2 weeks, during which patients should not take anything that could alter the clinical picture of the disease (systemic corticosteroids, antifungals, immunosuppressants).

**Statistical analysis**

Upon completion of the study, a randomization code will be opened. The Wilcoxon pairwise test will evaluate the effect of therapy in each of the groups. Between groups, the effect will be assessed by the Mann-Whitney test.

Patients will rate through 14 questions how often they have had mouth problems in the last month on a scale (Likert scale) of 0-4 (0-never, 1-almost never, 2-sometimes, 3-often, 4-always).

OHIP-CRO14:

1. Have you had or are having difficulty pronouncing words due to mouth problems?

2. Have you felt or feel unpleasant taste because of problems with your mouth?

3. Have you had or do you have severe mouth pain?

4. Have you been or are you uncomfortable eating a certain type of food due to mouth problems?

5. Have you thinking about your mouth?

6. Do you feel anxiety about mouth problems?

7. Do you find your diet unsatisfactory due to mouth problems?

8. Did you have to interrupt the meal due to mouth problems?

9. Do you find it harder to relax because of mouth problems?

10. Did you feel any discomfort due to mouth problems?

11. Have you been irritable towards others because of mouth problems?

12. Have you had problems performing daily tasks due to mouth problems?

13. Do you find that life gives you less satisfaction because of mouth problems?

14. Have you ever failed to function at all because of mouth problems?

Literature:

1. Nolan A, Baillie C, Badminton J et al. The efficacy of topical hyaluronic acid in the management of recurrent aphthous ulceration. J Oral Pathol Med 2006; 35: 461-5.
2. Khandwala A, Van Inwegen RG, Alfano MC. 5% amlexanox oral paste, a new treatment for recurrent minor aphthous ulcers: I. Clinical demonstration of acceleration of healing and resolution of pain. Oral Surg Oral Med Oral Pathol Oral Radiol Endod 1997; 83: 222-30.
3. Tyldesley WR, Harding SM. Betamethasone valerate aerosol in the treatment of oral lichen planus. Br J Dermatol 1997; 96: 659-62.
4. Zakrzewska JM, Chan ES-Y, Tornhill MH. A systematic review of placebo-controlled randomized clinical trials of treatments used in oral lichen planus. Br J Dermatol 2005; 153: 336-41.
5. Piboonniyom SO, Treister N, Pitiphat W et al. Scoring system for monitoring oral lichenoid lesions: A preliminary study. Oral Surg Oral Med Oral Pathol Oral Radiol Endod 2005; 99: 696-703.
6. Petricević N, Celebić A, Papić M et al. The Croatian version of the Oral Health Impact Profile Questionnaire. Coll Antropol 2009; 33: 841-7.
