# Supplementary material for: Topical NAVS naphthalan for the treatment of oral lichen planus and recurrent aphthous stomatitis: A double blind, randomized, parallel group study
Source: PLoS One. 2021 Apr 8;16(4):e0249862. doi: 10.1371/journal.pone.0249862 (PMC8031371; doi:10.1371/journal.pone.0249862)
Supplement: S4 File — (PDF) [file pone.0249862.s004.pdf]

SVEUČILIŠTE U ZAGREBU
FARMACEUTSKO-BIOKEMIJSKI FAKULTET
A. Kovačića 1, PP 156, 10001 ZAGREB, HRVATSKA

UNIVERSITY OF ZAGREB
FACULTY OF PHARMACY AND BIOCHEMISTRY
A. Kovačića 1, PP 156, 10001 ZAGREB, CROATIA

Prof.dr.sc. József Petrik, spec. med. biokemije
Zavod za medicinsku biokemiju i hematologiju
Domagojeva 2, 10000 Zagreb
Tel. + 385 6394 784
Fax. 4612 716
E-mail: jpetrik@pharma.hr

In vitro ispitivanje citotoksičnosti NAVS-a

Zagreb, 27. 09. 2010.

Istraživanje je provedeno za tvrtku MEDICOPHARMACIA d.o.o., Topniška 4, 1000 Ljubljana

SADRŽAJ

Sažetak

1. Opće informacije

2. Ispitivanje citotoksičnosti
2.1. Načelo određivanja citotoksičnosti MTT testom
2.2. Načelo određivanja citotoksičnosti mjerenjem katalitičke koncentracije laktat dehidrogenaze

3. Materijali i metode
3.1. Uzorak NAVS (niskoaromatski visokosteranski naftalan)
3.2. Kemikalije
3.3. Stanične linije (HaCaT CaCo -2 i A549 stanice te BJ-fibroblasti)
3.4. Postupak ispitivanja
3.5. Odabir koncentracija NAVS-a
3.6. Prosudba citotoksičnog djelovanja

4. Rezultati

5. Zaključak

6. Pohranjivanje podataka

Sažetak

Cilj ovog istraživanja bio je *in vitro* ispitivanje citotoksičnosti niskoaromatskog visokosteranskog naftalana (NAVS).

Istraživanje je provedeno na staničnim linijama HaCaT, Caco-2 i A549 te BJ fibroblastima. Stanice su kultivirane u hranjivom mediju DMEM (*Dulbecco's modified Eagle's medium*) uz 10% FBS, do 90% konfluentnosti te presađene u jažice mikrotitarske pločice gdje su tretirane s NAVS-om. Tretiranje je izvedeno paralelno na dva načina, uz površinski aktivne tvari (PAT) te uz dimetilsulfoksid (DMSO). Stanične linije su tretirane tijekom 24 i 48 sati s različitim koncentracijama NAVS-a priređenim uz PAT ili DMSO. Konačne koncentracije NAVS-a u kompletnom hranjivom mediju iznosile su: 0,1; (0,03); 0,01; 0,001; 0,0001 i 0,00001%. Tretiranje je također provedeno i s odgovarajućim koncentracijama PAT-a i DMSO-a.

Rezultati istraživanja pokazuju da NAVS ne djeluje citotoksično na tretirane HaCaT, Caco-2 i A549 stanice te BJ fibroblaste kada se uspoređuju rezultati s kontrolnim uzorcima uz odgovarajuće koncentracije PAT-a ili DMSO-a u mediju.

1. Opće informacije

1.1. Naručitelj ispitivanja: Doc. dr. sc. Ivan Alajbeg, Stomatološki fakultet Sveučilišta u Zagrebu

1.2. Izvođač:

Institucija: Farmaceutsko-biokemijski fakultet, Sveučilište u Zagrebu, A. Kovačića 1, 10000 Zagreb

Nositelj ispitivanja: Prof.dr.sc. József Petrik, spec. med. biokemije
Farmaceutsko-biokemijski fakultet
Sveučilište u Zagrebu
A. Kovačića 1, 10001, Zagreb
Zavod za medicinsku biokemiju i hematologiju
Domagojeva 2, 10000 Zagreb
Tel. + 385 6394 784
Fax. 4612 716
E-mail: jpetrik@pharma.hr

1.3. Oznaka uzorka: MPH03/10: NAVS (serija N°2)

1.4. Osoblje: **Nositelj ispitivanja:** Prof. dr. sc. József Petrik
Izvođenje ispitivanja: Prof. dr. sc. József Petrik
Dr. sc. Ivan Pepić
Prof. dr.sc. Roberta Petlevski
Ing. lab. med. Vesna Boričević

1.5. Datumi:

Zaprimanje uzorka:	01. 03. 2010.
Optimiranje uzoraka:	02. 03. 2010.
Optimiranje ispitivanja:	06. 03. 2010.
Početak ispitivanja:	26. 04. 2010.
Završetak ispitivanja:	18. 06. 2010.

1.6. Citotoksičnost NAVS-a bila je testirana MTT-testom i mjerenjem katalitičke koncentracije laktat dehidrogenaze u mediju iznad tretiranih HaCaT stanica i BJ-fibroblasta.

1.7.Izjava o autentičnosti:

Izjavljujem da se ispitivanje koje je predmet ovog izvješća provodilo u skladu sa ugovorom s klijentom i da izvješće sadrži točan opis dobivenih rezultata.

Nositelj ispitivanja:

Prof.dr.sc. József Petrik

____
Datum

2. Ispitivanje citotoksičnosti

Primjenom MTT testa određena je vijabilnost tretiranih i kontrolnih stanica *in vitro*. Pored toga, mjerenjem katalitičke aktivnosti laktat dehidrogenaze određen je prestanak vijabilnosti stanica.

2.1. Načelo određivanja citotoksičnosti MTT testom

MTT test je standardna kolorimetrijska metoda koja se koristi za mjerenje aktivnosti mikrosomalnih enzima dehidrogenaza. Dehidrogenaze reduciraju (cijepaju tetrazolni prsten) žuti, MTT [3-(4,5- dimetiltiazol-2-il)-2,5- difenil tetrazolijev bromid] u ljubičasti formazan. MTT je topiv u vodi a formazan nije te se pojavljuje u obliku ljubičastih kristala.

Stanična membrana živih stanica nepropusna je za kristale formazana, stoga tek nakon inkubacije i otapanja kristala formazana u DMSO uzorak postaje obojen. Absorbancija nastalog obojenja je mjera metaboličke aktivnosti, odnosno vijabilnosti stanica.

Nakon što se iz jažica sakupi medij za određivanje LDH, u svaku jažicu doda se MTT reagens. Stanice se zatim inkubiraju 3 sata na 37 °C uz 95% vlažnosti i 5% CO_2. Nakon inkubacije medij se odsiše i doda 200 µL otopine DMSO-a. Pločice za uzgoj stanica inkubiraju se 15 minuta na tresilici pri 600 okretaju u minuti da se kristalići formazana u potpunosti otope. Zatim se očitava apsorbancija pri 595 nm na čitaču mikrotitarskih pločica Victor 3 (Perkin Elmer). Rezultati metaboličke aktivnosti testiranih keratinocita, epitelnih stanica i fibroblasta (tretiranih sa: NAVS uz PAT, NAVS uz DMSO, PAT i DMSO) prikazuju se u odnosu na kontrolu.

2.2. Načelo određivanja citotoksičnosti mjerenjem katalitičke koncentracije laktat dehidrogenaze

Katalitička koncentracija LDH određuje se nakon tretiranja stanica tijekom 24 ili 48 sati na 37 °C uz 95% vlažnosti i 5% CO_2. Mediji (uzorci) iznad stanica se prenesu u centrifugirke. Uzorci se centrifugiraju 5 min na 400 x g. Supernatant se zatim odvoji i koristi se za određivanje katalitičke koncentracije LDH.

Praćenjem katalitičke koncentracije LDH u *in vitro* uvjetima moguće je procijeniti stupanj oštećenja stanične membrane, odnosno povećane vrijednosti enzima koreliraju s udjelom mrtvih stanica (nekroza, apoptoza uz sekundarnu nekrozu).

LDH katalizira u prisutnosti $NADH_2$ redukciju piruvata pri čemu nastaje laktat i NAD^+. Smanjenje apsorbancije odgovara prijelazu reduciranog koenzima u oksidirani oblik i mjera je aktivnosti enzima.

Katalitička aktivnost LDH mjerena je na 340 nm pri 37°C na analizatoru Trace.

3. Materijali i metode

3.1 Uzorak NAVS (niskoaromatski visokosteranski naftalan)
MPH03/10: NAVS (serija N°2)

Nanočestice NAVS + PAT u DMEM-u
Nanočestice PAT u DMEM-u
Otopina NAVS + DMSO u DMEM-u
Otopina DMSO u DMEM-u

3.2 Kemikalije

1. Kompletni medij: Dulbecco's modified Eagle's medium DMEM, (SIGMA–ALDRICH, D5671) obogaćen sa 10%-tnim serumom goveđeg fetusa, FBS (*engl. fetal bovine serum*), 2 mM glutamata, 100 U/mL penicilina, 100 µg/mL streptomicina i 0,25 µg/mL amfotericina, pripravljen je u sterilnim uvjetima.
2. Otopina fosfatnog pufera: 137 mM NaCl; 2,7 mM KCl; 1,4 mM KH2PO4, 4,3 mM Na2HPO4 x 7 H2O; sterilno filtrirano
3. 0,05% tripsin-EDTA, (GIBCO)
4. Matična otopina MTT reagens (SIGMA–ALDRICH)
5. Dimetilsulfoksid, DMSO (SIGMA–ALDRICH)
6. Reagens za određivanje LDH (HERBOS Dijagnostika d.o.o.)

3.3 Stanične linije

Istraživanje je provedeno na staničnim linijama:
HaCaT (human keratinocyte cell line)
Caco-2 (human epithelial colorrectal adenocarcinoma cells)
A549 (adenocarcinomic human alveolar epithelial cells)
BJ fibroblastima (human foreskin fibroblasts BJ)

3.4 Postupak ispitivanja

HaCaT, Caco-2 i A549 stanice te BJ fibroblasti kultivirani su u kompletnom DMEM mediju. Nakon što su stanice postigle 90% konfluentnosti, sa adheriranih stanica odsiše se medij i stanice se jednokratno isperu sa 5 mL PBS-a. PBS se odsiše i doda se 1 mL 0,05% otopine tripsina kako bi se stanice odvojile od podloge. Nakon tripsinizacije dodaje se odgovarajući kompletni medij i slijedi resuspendiranje stanica. Nasadi se 10000 stanica u 200 µL stanične suspenzije u jažice na mikrotitarsku pločicu za uzgoj stanica. Stanice se inkubiraju na 37 °C uz 95% vlažnosti i 5% CO_2 do 90% konfluentnosti. Konfluentnost se provjerava inverzinim mikroskopom. Stanice se tretiraju definiranim koncentracijama NAVS-a, priređenim uz PAT ili DMSO, površinski aktivnih tvari (PAT) i dimetilsulfoksida (DMSO) tijekom 24 i 48 sati.

3.5 Odabir koncentracija NAVS-a

Koncentracije NAVS-a, PAT-a i DMSO-a su odabrane na temelju rezultata preliminarnih ipitivanja i optimiranja aplikacije NAVS-a u *in vitro* uvjetima. Stanične linije su tretirane tijekom 24 i 48 sati s različitim koncentracijama NAVS-a priređenim uz PAT ili DMSO (konačne koncentracije NAVS-a u kompletnom hranjivom mediju iznosile su: 0,1; 0,01; 0,001; 0,0001 i 0,00001%). Tretiranje je provedeno i s odgovarajućim koncentracijama PAT-a i DMSO-a.

TRETIRANJE STANICA I OZNAČAVANJE UZORAKA:

HaCaT stanice i BJ fibroblasti su tretirani koncentracijama navedenim u ***tablici 1*** tijekom 24 i 48 sati. (NAVS : DMSO = 1 : 1; NAVS : PAT = 1 : 1)

Tablica 1. Koncentracije NAVS-a; PAT-a i DMSO-a tijekom tretiranja stanica.

**Kontrola**	**PAT**	**DMSO**	**NAVS+PAT**	**NAVS+DMSO**
**K**	**P1**<br>0,1%	**D1**<br>0,1%	**NP1**<br>0,1%	**ND1**<br>0,1%
	**P2**<br>0,01%	**D2**<br>0,01%	**NP2**<br>0,01%	**ND2**<br>0,01%
	**P3**<br>0,001%	**D3**<br>0,001%	**NP3**<br>0,001%	**ND3**<br>0,001%
	**P4**<br>0,0001%	**D4**<br>0,0001%	**NP4**<br>0,0001%	**ND4**<br>0,0001%
	**P5**<br>0,00001%	**D5**<br>0,00001%	**NP5**<br>0,00001%	**ND5**<br>0,00001%

Oznake uzoraka: **K** = kontrola, **P1-P5** = PAT u navedenim koncentracijama, **D1-D5** = DMSO u navedenim koncentracijama **NP1-NP5** = NAVS+PAT u navedenim koncentracijama i **ND1-ND5** = NAVS+DMSO u navedenim koncentracijama.

CaCo-2 i A549 stanice su tretirane koncentracijama nevedenim u ***tablici 2*** tijekom 24 i 48 sati. (NAVS : DMSO = 1 : 1; NAVS : PAT = 1 : 3,3)

Tablica 2. Koncentracije NAVS-a; PAT-a i DMSO-a tijekom tretiranja stanica.

**Kontrola**	**PAT**	**DMSO**	**NAVS+PAT**	**NAVS+DMSO**
**K**<br>0,00%	**P1**<br>0,1%	**D1**<br>0,1%	**NP1**<br>0,03%+<br>0,1%	**ND1**<br>0,1% +<br>0,1%
	**P2**<br>0,033%	**D2**<br>0,01%	**NP2**<br>0,01%+<br>0,033%	**ND2**<br>0,01% +<br>0,01%
	**P3**<br>0,0033%	**D3**<br>0,001%	**NP3**<br>0,001% +<br>0,0033%	**ND3**<br>0,001% +<br>0,001%
	**P4**<br>0,00033%	**D4**<br>0,0001%	**NP4**<br>0,0001%+<br>0,00033%	**ND4**<br>0,0001% +<br>0,0001%
	**P5**<br>0,000033%	**D5**<br>0,00001%	**NP5**<br>0,00001%+<br>0,000033%+	**ND5**<br>0,00001% +<br>0,00001%

Oznake uzoraka: **K** = kontrola, **P1-P5** = PAT u navedenim koncentracijama, **D1-D5** = DMSO u navedenim koncentracijama **NP1-NP5** = NAVS+PAT u navedenim koncentracijama i **ND1-ND5** = NAVS+DMSO u navedenim koncentracijama.

3.6 Prosudba citotoksičnog djelovanja NAVS-a

Mogući citotoksični učinak NAVS-a će se procijeniti uspoređivanjem rezultata MTT testa i katalitičkih koncentracija LDH prema odgovarajućim kontrolnim uzorcima uz PAT ili DMSO.

4. Rezultati

Rezultati MTT testa - numeričke vrijednosti predstavljaju srednje vrijednosti apsorbancije pri 595 nm na temelju 8 paralelnih određivanja.

MTT1 / PAT / NAVS+PAT / HaCaT / 24h / 48h

	**24 h**	**48h**
**K**	0,531	0,415
**P1**	0,321	0,296
**NP1**	0,347	0,325
**P2**	0,391	0,324
**NP2**	0,360	0,358
**P3**	0,413	0,391
**NP3**	0,447	0,392
**P4**	0,422	0,384
**NP4**	0,467	0,395
**P5**	0,464	0,383
**NP5**	0,489	0,410

MTT2 / DMSO / NAVS+DMSO / HaCaT / 24h / 48h

	**24 h**	**48h**
**K**	0,627	0,646
**D1**	0,442	0,487
**ND1**	0,390	0,463
**D2**	0,435	0,534
**ND2**	0,450	0,549
**D3**	0,447	0,551
**ND3**	0,522	0,542
**D4**	0,612	0,601
**ND4**	0,592	0,583
**D5**	0,615	0,605
**ND5**	0,623	0,609

Rezultati MTT testa - numeričke vrijednosti predstavljaju srednje vrijednosti apsorbancije pri 595 nm na temelju 8 paralelnih određivanja.

MTT3 / PAT / NAVS+PAT / BJ fibroblasti / 24h / 48h

	**24 h**	**48h**
**K**	0,286	0,298
**P1**	0,203	0,157
**NP1**	0,205	0,155
**P2**	0,233	0,156
**NP2**	0,221	0,148
**P3**	0,254	0,276
**NP3**	0,261	0,239
**P4**	0,283	0,269
**NP4**	0,285	0,288
**P5**	0,291	0,291
**NP5**	0,304	0,267

MTT4 / DMSO / NAVS+DMSO / BJ fibroblasti / 24h / 48h

	**24 h**	**48h**
**K**	0,285	0,276
**D1**	0,225	0,175
**ND1**	0,206	0,170
**D2**	0,221	0,187
**ND2**	0,222	0,181
**D3**	0,244	0,236
**ND3**	0,230	0,229
**D4**	0,258	0,233
**ND4**	0,224	0,237
**D5**	0,280	0,259
**ND5**	0,269	0,249

Rezultati MTT testa - numeričke vrijednosti predstavljaju srednje vrijednosti apsorbancije pri 595 nm na temelju 8 paralelnih određivanja.

MTT5 / PAT / NAVS+PAT / Caco-2/ 24h / 48h

	**24 h**	**48h**
**K**	1,349 ± 0,153	1,398 ± 0,126
**P1**	1,067 ± 0,196	0,877 ± 0,123*
**NP1**	1,051 ± 0,163	0,912 ± 0,143*
**P2**	1,168 ± 0,151	1,252 ± 0,102
**NP2**	1,241 ± 0,098	1,260 ± 0,095
**P3**	1,239 ± 0,140	1,377 ± 0,130
**NP3**	1,203 ± 0,111	1,342 ± 0,097
**P4**	1,350 ± 0,122	1,401 ± 0,110
**NP4**	1,365 ± 0,097	1,355 ± 0,162
**P5**	1,274 ± 0,115	1,297 ± 0,115
**NP5**	1,314 ± 0,099	1,365 ± 0,136

MTT6 / DMSO / NAVS+DMSO / Caco-2 / 24h / 48h

	**24 h**	**48h**
**K**	1,267 ± 0,079	1,485 ± 0,186
**D1**	1,104 ± 0,129	1,188 ± 0,124
**ND1**	1,165 ± 0,082	1,241 ± 0,095
**D2**	1,202 ± 0,083	1,385 ± 0,163
**ND2**	1,235 ± 0,113	1,379 ± 0,203
**D3**	1,314 ± 0,117	1,429 ± 0,126
**ND3**	1,320 ± 0,070	1,315 ± 0,213
**D4**	1,269 ± 0,105	1,513 ± 0,089
**ND4**	1,341 ± 0,137	1,465 ± 0,126
**D5**	1,273 ± 0,096	1,477 ± 0,164
**ND5**	1,327 ± 0,124	1,501 ± 0,221

Rezultati MTT testa - numeričke vrijednosti predstavljaju srednje vrijednosti apsorbancije pri 595 nm na temelju 8 paralelnih određivanja.

MTT7 / PAT / NAVS+PAT / A549 / 24h / 48h

	**24 h**	**48h**
**K**	2,375 ± 0,269	2,471 ± 0,213
**P1**	2,012 ± 0,194	1,530 ± 0,266*
**NP1**	2,152 ± 0,216	1,598 ± 0,162*
**P2**	2,278 ± 0,098	2,239 ± 0,243
**NP2**	2,224 ± 0,115	2,342 ± 0,316
**P3**	2,273 ± 0,316	2,480 ± 0,321
**NP3**	2,260 ± 0,138	2,453 ± 0,164
**P4**	2,367 ± 0,310	2,466 ± 0,217
**NP4**	2,326 ± 0,211	2,502 ± 0,206
**P5**	2,497 ± 0,219	2,483 ± 0,303
**NP5**	2,433 ± 0,159	2,407 ± 0,438

MTT8 / DMSO / NAVS+DMSO / A549 / 24h / 48h

	**24 h**	**48h**
**K**	2,584 ± 0,165	2,694 ± 0,205
**D1**	2,636 ± 0,089	2,556 ± 0,119
**ND1**	2,495 ± 0,106	2,584 ± 0,098
**D2**	2,463 ± 0,095	2,562 ± 0,083
**ND2**	2,572 ± 0,090	2,623 ± 0,075
**D3**	2,561 ± 0,206	2,589 ± 0,233
**ND3**	2,559 ± 0,112	2,629 ± 0,156
**D4**	2,313 ± 0,180	2,601 ± 0,138
**ND4**	2,538 ± 0,129	2,650 ± 0,152
**D5**	2,595 ± 0,116	2,657 ± 0,196
**ND5**	2,612 ± 0,173	2,716 ± 0,125

Rezultati određivanja katalitičke koncentracije laktat dehidrogenaze - numeričke vrijednosti predstavljaju srednje vrijednosti U/L na temelju 3 paralelna određivanja.

LDH 1 / PAT / NAVS+PAT / HaCaT / 24h / 48h

	**24 h**	**48h**
**K**	36	26
**P1**	318	317
**NP1**	298	304
**P2**	147	189
**NP2**	148	212
**P3**	176	242
**NP3**	214	217
**P4**	221	215
**NP4**	188	229
**P5**	223	190
**NP5**	157	152

LDH 2 / DMSO / NAVS+DMSO / HaCaT / 24h / 48h

	**24 h**	**48h**
**K**	21	29
**D1**	111	129
**ND1**	122	130
**D2**	61	87
**ND2**	72	111
**D3**	32	49
**ND3**	41	55
**D4**	36	38
**ND4**	41	33
**D5**	25	29
**ND5**	37	36

Rezultati određivanja katalitičke koncentracije laktat dehidrogenaze - numeričke vrijednosti predstavljaju srednje vrijednosti U/L na temelju 3 paralelna određivanja.

LDH 3 / PAT / NAVS+PAT / BJ fibroblasti / 24h / 48h

	**24 h**	**48h**
**K**	10	8
**P1**	65	74
**NP1**	67	77
**P2**	31	19
**NP2**	31	17
**P3**	15	17
**NP3**	20	16
**P4**	13	30
**NP4**	16	22
**P5**	16	17
**NP5**	6	18

LDH 4 / DMSO / NAVS+DMSO / BJ fibroblasti / 24h / 48h

	**24 h**	**48h**
**K**	4	6
**D1**	22	73
**ND1**	18	67
**D2**	15	60
**ND2**	13	56
**D3**	15	39
**ND3**	14	19
**D4**	11	17
**ND4**	15	22
**D5**	9	11
**ND5**	11	12

Rezultati određivanja katalitičke koncentracije laktat dehidrogenaze - numeričke vrijednosti predstavljaju srednje vrijednosti U/L na temelju 3 paralelna određivanja.

LDH 5 / PAT / NAVS+PAT / Caco-2/ 24h / 48h

	**24 h**	**48h**
**K**	29	74
**P1**	67	166
**NP1**	54	149
**P2**	49	114
**NP2**	48	121
**P3**	42	105
**NP3**	47	89
**P4**	41	84
**NP4**	37	85
**P5**	36	94
**NP5**	33	85

LDH 6 / DMSO / NAVS+DMSO / Caco-2 / 24h / 48h

	**24 h**	**48h**
**K**	45	62
**D1**	74	86
**ND1**	68	78
**D2**	65	82
**ND2**	67	80
**D3**	61	74
**ND3**	59	75
**D4**	55	79
**ND4**	52	69
**D5**	53	72
**ND5**	50	66

Rezultati određivanja katalitičke koncentracije laktat dehidrogenaze - numeričke vrijednosti predstavljaju srednje vrijednosti U/L na temelju 4 paralelna određivanja.

LDH 7 / PAT / NAVS+PAT / A549 / 24h / 48h

	**24 h**	**48h**
**K**	14	66
**P1**	23	75
**NP1**	27	87
**P2**	18	74
**NP2**	12	77
**P3**	26	80
**NP3**	34	78
**P4**	40	93
**NP4**	39	107
**P5**	60	136
**NP5**	77	155

LDH 8 / DMSO / NAVS+DMSO / A549 / 24h / 48h

	**24 h**	**48h**
**K**	26	46
**D1**	31	47
**ND1**	35	53
**D2**	29	45
**ND2**	29	47
**D3**	27	47
**ND3**	28	48
**D4**	25	44
**ND4**	31	46
**D5**	28	44
**ND5**	32	51

5. Zaključak

Prema rezultatima ispitivanja vijabilnosti HaCaT i BJ fibroblasta može se zaključiti da koncentracije NAVS-a manje od 0,001% (uz DMSO ili PAT) ne utječu na metaboličku aktivnost testiranih stanica.

Veće koncentracije NAVS-a, ovisno o dozi, smanjuju broj živih HaCaT stanica i BJ fibroblasta za približno 15%, odnosno 30% za navedene stanične linije. Važno je naglasiti da PAT i DMSO bez NAVS-a također djeluju vrlo slično na broj živih stanica, tj. smanjuju metaboličku aktivnost u tretiranim uzorcima. Primjećeno je da PAT u odnosu na DMSO djeluje više citotoksično, ali u kombinaciji s NAVS-om i prilikom usporedbe s uzorcima tretiranim samo s PAT, NAVS smanjuje citotoksično djelovanje PAT-a.

Može se zaključiti da NAVS u usporedbi s odgovarajućim kontrolnim uzorcima (PAT ili DMSO) ne pokazuje citotoksično djelovanje kod HaCaT stanica te kod BJ fibroblasta.

Prema rezultatima ispitivanja vijabilnosti Caco-2 i A549 stanica, može se zaključiti da koncentracije NAVS-a od 0,03% (uz PAT) statistički značajno utječu na metaboličku aktivnost kod obije vrste stanica tretiranih tijekom 48 sati. Međutim, ako se vrijednosti uspoređuju prema uzorcima uz odgovarajuće količine PAT-a, može se zaključiti da citotoksični učinak pripada PAT-u a ne NAVS-u.

Općenito, praćenje citotoksičnog učinka mjerenjem katalitičke koncentracije laktat dehidrogenaze manje je osjetljivo od MTT testa. Međutim, dobiveni rezultati su u skladu s rezultatima MTT testova, gdje upravo emulgatori (PAT ili DMSO) pokazuju citotoksične učinke. Uspoređivanjem katalitičke aktivnosti LDH također se može zaključiti da se citotoksično djelovanje odnosi na PAT ili DMSO, a ne na NAVS.

Uspoređivanjem dobivenih rezultata za NAVS+PAT ili za NAVS+DMSO s uzorcima uz odgovarajuće koncentracije PAT-a ili DMSO-a u mediju, može se zaključiti, da NAVS ne djeluje citotoksično na tretirane HaCaT, Caco-2 i A549 stanice te BJ fibroblaste.

6. Pohranjivanje podataka

Izvorni podaci će se pohranjivati tijekom narednih 5 godina na Zavodu za medicinsku biokemiju i hematologiju Farmaceutsko–biokemijskog fakulteta Sveučilišta u Zagrebu.
