# Supplementary material for: Topical NAVS naphthalan for the treatment of oral lichen planus and recurrent aphthous stomatitis: A double blind, randomized, parallel group study
Source: PLoS One. 2021 Apr 8;16(4):e0249862. doi: 10.1371/journal.pone.0249862 (PMC8031371; doi:10.1371/journal.pone.0249862)
Supplement: S5 File — (PDF) [file pone.0249862.s005.pdf]

IZVJEŠĆE

Predklinička toksikološka studija od 7 dana prema OECD Guideline od 1995. godine koja traje 28 dana.

Ispitivana supstanca: NAVS (u daljnjem tekstu **NAVS)**

Pripravak NAVS: priređen je od strane: doc. dr. sc. Ivan Alajbeg, Stomatološki fakultet

Predmet istraživanja: utvrditi štetnost primjene pripravka NAVS *per os* kroz 7 dana

Pripravak je testiran na miševima soja $C_{57}Bl$, oralna primjena (sondiranje), kroz period od 7 dana.

Test-sistem

- Miševi, $C_{57}Bl(H\text{-}2^b)$, iz uzgoja Instituta Ruđer Bošković, Zagreb, Hrvatska
- Miševi su se prilagođavali na uvjete prostorije, u kojoj su držani za vrijeme trajanja pokusa, tjedan dana prije početka pokusa
- težina i starost miševa na početku pokusa: $C_{57}Bl$ – prosječno 24 g (u rasponu od 22 do 26 g); starosti 3.0 mjeseca
- od okota do početka ispitivanja životinje su držane u standardnim uvjetima (prema GLP standardima): 20 miševa po kavezu, 12/12 sati dan/ noć, temp. 22˚C-24˚C, vlažnost 50-70%, hranjeni standardnom laboratorijskom hranom Mucedola , Settino Milanese, Italy, prema GLP standardima , *ad libitum*
- za vrijeme akutnog ispitivanja u kavezima su držana po 3 miša

Doza NAVS-a i skupine

2.1. Eksperimentalne grupe

Grupe	oznaka	broj miševa (n)	doza (mg/kg)
1	Kontrola	6	0
2	NAVS	6	100 (jednokratno)
3	NAVS	6	1000 (jednokratno)
4	NAVS	6	2000 (jednokratno)
5	Kontrola	6	0 (sonda 7 dana)
6	NAVS	6	2000 (sondom 7 dana)

Akutno toksičnost – prvi dio od 7 dana

- Težina tijela u trenutku žrtvovanja
- Količina pojedene hrane nakon 7-og dana
- Prosječni volumen urina nakon 7-og dana

Analiza urina : Combur 10Test M, Roche Diagnostic GmbH, Germany

Glukoza (g/dL)
Bilirubin
Specifična težina (g/L)
Eritrociti br./µL
pH
Proteini (mg/dl)
Urobilinogen (mg/dl)
Nitriti
Leukociti (br./µl)

Analiza krvi: Hematološki parametri su analizirani pomoću: Advia 60 blood cell counter (Siemens Medical Diagnostics, USA)

- broj eritrocita
- leukociti
- limfociti
- hemoglobina
- hematokrit

Analiza seruma: Kliničko-kemijski parametri su određeni pomoću: Olympus Au600 (Olympus Optical Co., Japan)

Alkalna fosfataza
Glukoza
AST
ALT
Bilirubin
Kolesterol
Trigliceridi
Fosfat
Kalcij

Ponašanje životinja - praćene su dnevno

REZULTATI

Trajanje ispitivanja

Početak: 24. kolovoz 2010. god.
Završetak: 31. Kolovoz 2010.

Tablica 1. Masa tijela, količina pojedene hrane te volumen izlučene mokraće tijekom 24 sata boravka u metaboličkom kavezu (vrijednosti izražene po mišu)

**Grupe**	**Masa tijela (g)***	**Težina hrane (g)***	**Volumen urina (ml)***
**1**	24,8±0,8	3,1±0,3*	0,5±0,2
**2**	24,4±0,5	2,8±0,4	0,6±0,3
**3**	24,8±0,5	2,9±0,5	0,5±0,1
**4**	24,5±0,6	3,0±0,3	0,7±0,3
**5**	22,8±0,5	3,1±0,2	0,6±0,2
**6**	23,2±0,9	2,9±0,2	0,5±0,3

*srednja vrijednost ± **s**tandardna devijacija

Table 2. Analiza urina

**Grupe**	**Parametri**								
	**Glukoza (g/dL)**	**Bilirubin**	**Specifična težina (g/L)**	**Eritrociti br./µL**	**pH**	**Proteini (mg/dl)**	**Urobilinogen (mg/dl)**	**Nitriti**	**Leukociti (br./µl)**
**1**	neg	normal	1.020	neg	6,.5	neg	norm	neg	Neg
**2**	neg	neg	1.015	neg	6,5	neg	norm	neg/poz	Neg
**3**	neg	neg	1.025	neg	6,0	neg	norm	neg	+/-
**4**	neg	neg	1.025	neg	6,5	neg	norm	neg	Neg
**5**	neg	neg	1,015	neg	6,5	neg	norm	neg	Neg
**6**	neg	neg	1,025	neg	6,0	+/-	norm	neg	Neg

Tablica 3. Hematološki parametri u uzorcima krvi

**Grupe**	**Parametri**				
	**Eritrociti*** 10^{12}/L	**Leukociti*** 10^{9}/L	**Limfociti** %	**Hemoglobin*** g/L	**Hematokrit*** L/L
**1**	4,7±0,3	3,8±1,2	88	89±11,4	0,22±0,1
**2**	4,4±0,3	4,8±0,8	89	89±6,2	0,26±0,09
**3**	5,1±0,2	4,2±0,3	92	85±4,4	0,24±0,07
**4**	4,8±0,2	4,7±1,4	90	99±6,9	0,21±0,08
**5**	3,6±0,1	3,6±0,6	92	89±5,3	0,23±0,08
**6**	4,5±0,2	4,3±0,3	91	91±3,1	0,24±0,09

**srednja vrijednost ± standardna devijacija

Table 4. Kliničko-kemijski parametri u serumu miševa

Grupe	Parametri								
	AF U/L	Glukoza Mm/L	AST U/L	ALT U/L	Bilirubin µM/L	Chol	Trig	Anorganski P mM/L	Ca mM/L
**1**	104±0,5	6,6±0,9	121±13,5	48±4,5	1±0,0	1,9±0,005	1,4±0,12	2,85±0,35	2,34±0,09
**2**	115±8,5	3,8±0,4	166±20,5	60±8	1±0,0	1,61±0,18	1,21±0,02	2,92±0,07	2,29±0,2
**3**	131±0,5	3,55±0,1	175±12	52,5±2,5	1,5±0,5	1,79±0,04	1,01±0,03	2,82±0,12	2,195±0,04
**4**	130±10,0	4,25±0,4	128±3,0	55±5,.5	1,5±0,5	1,76±0,06	1,075±0,05	2,67±0,095	2,165±0,02
**5**	151±4,0	4,55±0,9	130±24,5	59,5±8,5	2,5±0,5	1,64±0,09	1,045±0,12	2,45±0,23	2,14±0,04
**6**	122±3,0	6,0±0,4	141±9,0	55±4,5	2±0,0	1,91±0,07	1,49±0,8	2,29±0,2	2,16±0,03

Tjelesna masa - Tablica (I)

C57Bl miševi
nema razlike između ispitivane i kontrolne skupine

Količina popijene vode - Tablica (I)

nema razlike između ispitivane i kontrolne skupine

Količina izlučenog urina - Tablica (I)

nema razlike između ispitivane i kontrolne skupine

Analiza urina - Tablica (II)

Svi ispitani parametri su u rasponu kontrolnih vrijednosti

Hematološki parametri -Tablica (III)

Svi parametri su u granicama normale

Kliničko-kemijski parametri - Tablica (IV)

Svi izmjereni parametri su granicama kontrolnih vrijednosti i međusobno se ne razlikuju

Patološka analiza
- nije rađena, ali organi jetra, slezena, bubreg i pankreas su pospremljeni za eventualnu histološku analizu

Smrtnost životinja

- tijekom akutnog i subkroničnog toksikološkog ispitivanja od 7 dana niti jedna životinja nije uginula

Zaključak

Oralno (sondom) davanje NAVS-a miševima soja $C_{57}Bl$ kroz period od 7 dana nije prouzročio nikakve promjene koje bi mogli smatrati rezultatom ispitivanja.

U Zagrebu, 7 rujna 2010. Godine

Voditelj laboratorija LMET

Dr.sc. Mirko Hadžija
