# Supplementary material for: Topical NAVS naphthalan for the treatment of oral lichen planus and recurrent aphthous stomatitis: A double blind, randomized, parallel group study
Source: PLoS One. 2021 Apr 8;16(4):e0249862. doi: 10.1371/journal.pone.0249862 (PMC8031371; doi:10.1371/journal.pone.0249862)
Supplement: S6 File — (PDF) [file pone.0249862.s006.pdf]

# Određivanje mutagenog potencijala PY Naftalana Ames bakterijskim testom

## Stručno izvješće

| | |
|---|---|
| Naručitelj: | **Doc. dr. sc. Ivan Alajbeg,** Zavod za oralnu medicinu, Gundulićeva 5 HR-1000 Zagreb |
| Narudžba | **Prema Narudžbi od 09. ožujka 2009. godine** |
| Izvršitelj: | **Laboratorij za molekularnu ekotoksikologiju,** Zavod za istraživanje mora i okoliša, Institut „Ruđer Bošković“, Bijenička cesta 54, HR-1000 Zagreb |



| | |
|---|---|
| **Koordinator analize i autor stručnog izvješća:** | |
| **Dr. sc. Tvrtko Smital**, viši znanstveni suradnik, voditelj Laboratorija | Laboratorij za molekularnu ekotoksikologiju, Zavod za istraživanje mora i okoliša, Institut „Ruđer Bošković“ u Zagrebu |
| **Suradnik i izvršitelj analize:** | |
| **Mr. sc. Roberta Sauerborn Klobučar** | Laboratorij za molekularnu ekotoksikologiju, Zavod za istraživanje mora i okoliša, Institut „Ruđer Bošković“ u Zagrebu |



U Zagrebu, 25. ožujka 2009. godine

Voditelj Laboratorija za molekularnu ekotoksikologiju:

______________________________

dr. Tvrtko Smital

Predstojnik Zavoda za istraživanje mora i okoliša:

______________________________

dr. Tarzan Legović

v.d. ravnatelj Instituta "Ruđer Bošković":

______________________________

dr. Jaroslav Horvat

**Dr. sc. Tvrtko Smital**
Laboratorij za molekularnu ekotoksikologiju
Zavod za istraživanje mora i okoliša
Institut Ruđer Bošković
Bijenička 54, 10000 Zagreb
Tel: 01/45 61 088
Fax: 01/46 80 243
E-mail: smital@irb.hr

# STRUČNO IZVJEŠĆE

za predmet:

**- Određivanje mutagenog potencijala PY Naftalana Ames bakterijskim testom -**

## UVOD

Kako bi se utvrdio mogući mutageni učinak složene mješavine ugljikovodika deklarirane kao „PY Naftalan", na traženje Naručitelja izvršena je laboratorijska analiza mutagenog potencijala uzorka PY Naftalana međunarodno priznatom metodom, Ames bakterijskim testom na mutagene.

## MATERIJAL I METODE

### Priprema uzorka

Uzorak od oko 500 ml otopine svijetložute boje, deklariran od strane Naručitelja kao PY Naftalan, dostavljen je u Laboratorij za molekularnu ekotoksikologiju (LME) u staklenoj boci, te je odmah pohranjen u hladnjak (+4°C), gdje je držan do izvršenja analiza. Pored uzorka Naručitelj nam je dostavio i relevantne znanstvene publikacije u kojima su obrađivane kemijske karakteristike i/ili biološka aktivnost PY Naftalana. S obzirom da se očigledno radi o kompleksnoj smjesi ugljikovodika, prije izvođenja samog Ames testa napravljene su preliminarne analize usmjerene na testiranje:

- topivosti PY naftalana u dimetil sulfoksidu (DMSO) koji se uobičajeno u Ames testu koristi kao medij za otapanje/razrjeđivanje kompleksnih uzoraka;
- eventualne kronične toksičnosti PY Naftalana za bakterijske sojeve TA98 i TA100 vrste *Salmonella typhimurium*;
- aktivnosti i selektivnosti bakterijskih sojeva;
- metaboličkog potencijala S9 frakcije jetre štakora i.p. tretiranog Aroklorom 1254 (500 mg/kg) koja je u Ames testu korištena kao aktivacijski sustav za detekciju premutagena.

Preliminarne analize pokazale su prihvatljivu topivost uzorka PY Naftalana u DMSO-u, te izostanak toksičnog učinka koji bi onemogućio vjerodostojno korištenje Ames testa. Inicijalna karakterizacija bakterijskih sojeva bila je pozitivna, a aktivacijski potencijal S9 frakcije bio je visok. U skladu s tim ulaznim parametrima ocijenili smo da se može provesti vjerodostojno određivanje (pre)mutagenog potencijala PY Naftalana korištenjem TA98 i TA100 sojeva *S. typhimurium*, prema klasičnom Amest test protokolu.

**Ames test**

Ames test na mutagenost izvođen je prema metodi Maron i Ames (1983.; Mortelmans i Zeiger, 2000) s TA98 i TA100 sojevima *S. typhimurium*. Test je proveden korištenjem dva bakterijska soja kako bi se mogle detektirati mutacije koje uzrokuju pomak okvira čitanja (TA98), odnosno mutacije koje uzrokuju zamjenu parova baza u DNA (TA100). Testiranje je izvršeno s nerazrjeđenom tvari, te sa serijom razrjeđenja u širokom rasponu (volumni omjeri 1:1, 1:5, 1:10, 1:50, 1:100, 1:500 i 1:1000). Uzorak PY Naftalana pri tome je razrjeđivan DMSO-om.

Prilikom izvođenja testa obuhvaćene su i sve pozitivne i negativne kontrole, u svim navedenim slučajevima sa i bez metaboličkog aktivacijskog sustava (+, odnosno – S9 mix). Kao pozitivne kontrole korišteni su poznati izravni i neizravni (premutageni) mutageni spojevi:

- 4-Nitro-o-fenildiamin (NOPD) kao modelni izravni mutagen (20 μg/ploči); te
- benzo(a)piren (BaP) kao modelni premutagen (1 μg/ploči).

Negativne kontrole:

- ploče bez bakterija;
- kontrola broja spontanih revertanata (bez dodatka modelnih (pre)mutagena ili uzorka);
- kontrola otapala (DMSO).

Svi uzorci testirani su u triplikatu.

**Literatura**

D. Maron, B.N. Ames, Revised methods for the *Salmonella* mutagenicity test, Mutat. Res. 113 (1983) 173–215.

K. Mortelmans, E. Zeiger, The Ames *Salmonella*/microsome mutagenicity assay, Mutat. Res. 455 (2000) 29-60.

## REZULTATI

U Tablici 1. prikazani su rezultati Ames testa za PY Naftalan. Pri opisu rezultata prije svega je bitno znati da je temeljni kriterij za mutagenost u Amest testu broj pozitivnih (tzv. $His^+$) kolonija (revertanata) na ploči, koji u slučaju mutagene tvari mora biti najmanje dvostruko veći od broja spontanih revertanata, koji se točno zna za svaki soj *S. typhimurium* koji se koristi u Ames testu. U skladu s tim kriterijem modelni mutageni, odnosno premutageni koji se koriste kao kontrole postupka, moraju rezultirati brojem revertanata koji je uvjerljivo viši od broja spontanih revertanata. I u ovom slučaju mutageni odgovor koji se očekuje od modelnih spojeva dobro je poznat iz literature, i to opet za svaki soj *S. typhimurium*.

Iz rezultata prikazanih u **Tablici 1**. je prije svega jasno da je laboratorijski postupak bio proveden korektno:

- na pločama bez bakterija nije bilo spontanih revertanata koji bi ukazivali na zagađenje;
- broj spontanih revertanata bio je unutar očekivanih/preporučenih vrijednosti, tj. 20-40/ploči za TA98, te 100-200 za soj TA100;
- DMSO (100 µl/ploči) nije uzrokovao povećanje broja spontanih revertanata;
- NOPD (20 µg/ploči) je kao modelni izravni mutagen uzrokovao očekivano visoko povećanje broja revertanata (>1300 za TA98, te >1800 za soj TA100);
- BaP (1 µg/ploči) kao modelni premutagen nije uzrokovao povećanje broja TA100 revertanata bez aktivacijskog sustava (- S9 mix), dok je dodatak aktivacijskog sustava (+ S9 mix) rezultirao očekivano visokim (>500) povećanjem broja revertanata.

Nadalje, rezultati testiranja uzoraka PY naftalana jasno ukazuju da PY Naftalan nije niti u jednoj testiranoj koncentraciji/razrjeđenju uzrokovao dvostruko povećanje broja revertanata u odnosu na broj spontanih revertanata karakterističan za pojedini soj, i to bez i uz korištenje S9 enzimskog aktivacijskog sustava. Srednje vrijednosti broja revertanata za PY Naftalan kretale su se od 27-38 za soj TA98, odnosno 143-186 za TA100, što je unutar deklariranog raspona broja spontanih revertanata za TA98 (20-40), odnosno TA100 (100-200). Prema tome, ispitivani uzorak PY Naftalana ne može se prema kriterijima Ames testa smatrati mutagenim ili premutagenim.

**Tablica 1.** Rezultati analize mutagenog i premutagenog potencijala uzorka PY Naftalana Ames bakterijskim testom. Uzorak je testiran nerezrjeđen, te u naznačenom rasponu razrjeđenja, sa i bez metaboličkog aktivacijskog sustava (+, odnosno – S9 mix), kao što je to naznačeno u Materijalima i metodama. Rezultati predstavljaju brojeve pozitivnih ($His^+$) revertanata po test ploči, uz pripadajuće srednje vrijednosti i standardne devijacije (SD) uzoraka testiranih u triplikatu.

| ID uzorka | ***Salmonella typhimurium* - soj TA98** | | | | | | ***Salmonella typhimurium* - soj TA100** | | | | | |
|---|---|---|---|---|---|---|---|---|---|---|---|---|
| | **bez metaboličke aktivacije (- S9 mix)** | | | **s metaboličkom aktivacijom (+ S9 mix)** | | | **bez metaboličke aktivacije (- S9 mix)** | | | **s metaboličkom aktivacijom (+ S9 mix)** | | |
| | **br. His+ revertanata** | **Srednja vrijednost** | **SD** | **br. His+ revertanata** | **Srednja vrijednost** | **SD** | **br. His+ revertanata** | **Srednja vrijednost** | **SD** | **br. His+ revertanata** | **Srednja vrijednost** | **SD** |
| PY naftalan - nerazr. uzorak | 46<br>33<br>25 | **35** | 10,6 | 38<br>38<br>37 | **38** | 0,6 | 154<br>136<br>139 | **143** | 9,6 | 181<br>184<br>194 | **186** | 6,8 |
| PY naftalan - 1 : 1 | 27<br>28<br>25 | **27** | 1,5 | 41<br>33<br>36 | **37** | 4,0 | 163<br>140<br>141 | **148** | 13 | 158<br>167<br>135 | **153** | 16,5 |
| PY naftalan - 1 : 5 | 31<br>26<br>23 | **27** | 4,0 | 38<br>42<br>35 | **38** | 3,5 | 125<br>149<br>170 | **148** | 22,5 | 146<br>133<br>166 | **148** | 16,6 |
| PY naftalan - 1 : 10 | 35<br>27<br>23 | **28** | 6,1 | 32<br>28<br>29 | **30** | 2,1 | 137<br>130<br>167 | **145** | 19,7 | 149<br>166<br>137 | **151** | 14,6 |
| PY naftalan - 1 : 50 | 35<br>33<br>32 | **33** | 1,5 | 39<br>28<br>44 | **37** | 8,2 | 153<br>163<br>143 | **153** | 10,0 | 131<br>163<br>136 | **143** | 17,2 |
| PY naftalan - 1 : 100 | 26<br>31<br>30 | **29** | 2,6 | 41<br>33<br>34 | **36** | 4,4 | 155<br>142<br>156 | **151** | 7,8 | 142<br>144<br>147 | **144** | 2,5 |
| PY naftalan - 1 : 500 | 26<br>26<br>35 | **29** | 5,2 | 41<br>33<br>34 | **36** | 4,4 | 140<br>174<br>141 | **152** | 19,3 | 142<br>144<br>147 | **144** | 2,5 |
| PY naftalan - 1 : 1000 | 34<br>26<br>22 | **27** | 6,1 | 33<br>31<br>25 | **30** | 4,2 | 148<br>136<br>138 | **141** | 6,4 | 164<br>148<br>149 | **154** | 9,0 |
| **Kontrole postupka** | | | | | | | | | | | | |
| kontrola - bez bakterija | 0<br>0<br>0 | **0** | / | 0<br>0<br>0 | **0** | / | 0<br>0<br>0 | **0** | / | 0<br>0<br>0 | **0** | / |
| kontrola - spontani revertanti | 23<br>20<br>21 | **21** | 1,5 | 26<br>35<br>23 | **28** | 6,2 | 148<br>175<br>130 | **151** | 22,6 | 138<br>139<br>154 | **144** | 9,0 |
| kontrola - DMSO (100 µl/ploči) | 21<br>16<br>27 | **21** | 5,5 | 17<br>24<br>20 | **3,5** | 3,5 | 161<br>146<br>132 | **146** | 14,5 | 147<br>140<br>163 | **150** | 11,8 |
| kontrola - NOPD (20 µg/ploči) | > 1300<br>> 1300<br>> 1300 | **> 1300** | / | > 1300<br>> 1300<br>> 1300 | **> 1300** | / | > 1800<br>> 1800<br>> 1800 | **> 1800** | / | > 1800<br>> 1800<br>> 1800 | **> 1800** | / |
| kontrola - BaP (1 µg/ploči) | | | | | | | 170<br>133<br>162 | **155** | 19,5 | > 500<br>> 500<br>> 500 | **> 500** | / |

DMSO - dimetilsulfoksid
NOPD - 4-Nitro-o-fenildiamin
BaP - benzo(a)piren

## ZAKLJUČAK

U pogledu određivanja mutagenog, odnosno premutagenog potencijala ispitivanog uzorka PY Naftalana, na temelju dobivenih rezultata utvrdili smo da niti u jednom uzorku nije ustanovljeno prisustvo premutagenih i/ili mutagenih tvari, prema međunarodno prihvaćenim kriterijima za Ames bakterijski test. **U skladu s ovim rezultatima utvrđujemo da mješavina ugljikovodika deklarirana kao „PY naftalan“ nema mutageni ili premutageni učinak.**

Voditelj Laboratorija za molekularnu ekotoksikologiju:

______________________________

dr. sc. Tvrtko Smital
