# Supplementary material for: Topical NAVS naphthalan for the treatment of oral lichen planus and recurrent aphthous stomatitis: A double blind, randomized, parallel group study
Source: PLoS One. 2021 Apr 8;16(4):e0249862. doi: 10.1371/journal.pone.0249862 (PMC8031371; doi:10.1371/journal.pone.0249862)
Supplement: S7 File — (DOCX) [file pone.0249862.s007.docx]

Appendix 1

NAVS reduces the cytotoxic effect on surfactants (PAT). It can be concluded that NAVS does not show cytotoxic activity in HaCaT cells and in BJ fibroblasts compared to the corresponding control samples (PAT or dimethyl sulfoxide (DMSO)).

Appendix 2

Oral (gavage) administration of NAVS to mice of strain C_57_B1 during a period of 7 days did not cause any changes that could be considered as a test result.

Appendix 3

Regarding the determination of mutagenic or premutagenic potential of the tested sample of PY Naphthalan, based on the obtained results, we found that no sample was found to contain premutagenic and / or mutagenic substances, according to internationally accepted criteria for Ames bacterial test. In accordance with these results, we have established that the hydrocarbon mixture declared as “PY naphthalan” has no mutagenic or premutagenic effect.
